# Malaria Incidence Rates from Time Series of 2-Wave Panel Surveys

**DOI:** 10.1371/journal.pcbi.1005065

**Published:** 2016-08-10

**Authors:** Marcia C. Castro, Mathieu Maheu-Giroux, Christinah Chiyaka, Burton H. Singer

**Affiliations:** 1 Department of Global Health and Population, Harvard T. H. Chan School of Public Health, Boston, Massachusetts, United States of America; 2 Department of Infectious Disease Epidemiology, Imperial College London, London, United Kingdom; 3 Emerging Pathogens Institute, University of Florida, Gainesville, Florida, United States of America; 4 School of Social and Community Medicine, University of Bristol, Bristol, United Kingdom; Princeton University, UNITED STATES

## Abstract

Methodology to estimate malaria incidence rates from a commonly occurring form of interval-censored longitudinal parasitological data—specifically, 2-wave panel data—was first proposed 40 years ago based on the theory of continuous-time homogeneous Markov Chains. Assumptions of the methodology were suitable for settings with high malaria transmission in the absence of control measures, but are violated in areas experiencing fast decline or that have achieved very low transmission. No further developments that can accommodate such violations have been put forth since then. We extend previous work and propose a new methodology to estimate malaria incidence rates from 2-wave panel data, utilizing the class of 2-component mixtures of continuous-time Markov chains, representing two sub-populations with distinct behavior/attitude towards malaria prevention and treatment. Model identification, or even partial identification, requires context-specific a priori constraints on parameters. The method can be applied to scenarios of any transmission intensity. We provide an application utilizing data from Dar es Salaam, an area that experienced steady decline in malaria over almost five years after a larviciding intervention. We conducted sensitivity analysis to account for possible sampling variation in input data and model assumptions/parameters, and we considered differences in estimates due to submicroscopic infections. Results showed that, assuming defensible a priori constraints on model parameters, most of the uncertainty in the estimated incidence rates was due to sampling variation, not to partial identifiability of the mixture model for the case at hand. Differences between microscopy- and PCR-based rates depend on the transmission intensity. Leveraging on a method to estimate incidence rates from 2-wave panel data under any transmission intensity, and from the increasing availability of such data, there is an opportunity to foster further methodological developments, particularly focused on partial identifiability and the diversity of a priori parameter constraints associated with different human-ecosystem interfaces. As a consequence there can be more nuanced planning and evaluation of malaria control programs than heretofore.

## Author Summary

Incidence rates measure the transitions between the states of noninfected to infected per unit of time and per person at risk. Usually calculated from longitudinal observations, they provide an indication of how rapidly a disease develops in a population over time. In the context of malaria, longitudinal data on infection status are obtained through consecutive survey rounds, separated by a certain time interval. Depending on the length of the interval, some changes of infection status may be missed, and thus only uncensored information would be available. Methodology to calculate incidence rates from this type of data was first proposed in 1976, but its assumptions were not applicable to low transmission settings, particularly in the presence of control measures. No alternative methodology has been proposed in the past 40 years, limiting attempts to obtain estimates of incidence rates in the current scenario of declining malaria transmission worldwide. In this paper we address this gap and introduce new methodology to estimate malaria incidence rates from longitudinal data that can be applied to settings with any transmission level. We provide a complete example of the method, including sensitivity analysis, and an assessment of possible differences between data based on microscopy vs. PCR diagnostics. To facilitate replication and wide use of the method, we make available a programming code in R language and the example dataset.

## Introduction

Estimation of incidence, and in some cases recovery, rates for malaria infection is a central objective of ongoing community surveillance programs [1]. By *incidence rate* we mean the number of new infections acquired in a small interval of time per person at risk (i.e. uninfected) at the beginning of the interval. Analogously, a recovery rate is the number of terminations of infection in a small interval of time per person at risk (i.e. infected) at the beginning of the interval.

It is important to observe that *incidence rate*, as defined above, is not the same as *incidence*, as commonly used in the contemporary malaria literature [e.g., see reference 2]. In the latter case, *incidence* is defined as [Number of positive species-specific clinical cases observed during the duration of a survey]/[(Number of people observed over the survey duration) * (duration of survey)]. We quantitatively compare and contrast these notions in the Discussion section.

Ideally one would like to have continuous histories of infection status on designated populations so that incidence rates could be directly ascertained over short intervals starting at any designated time. This would facilitate showing the impact of local-in-time weather events, seasonal variation, and the impact of intervention strategies on these rates at arbitrary times of interest to health service workers, government personnel setting malaria control policy, and research investigators.

Continuous infection status histories are virtually never available at a community level. The most common longitudinal data collection plan is a time series of 2-wave panel data sets (or observations of infection status on the same individuals taken at two time points separated by an interval of length Δ), with spacing between waves varying from a few weeks [3,4] to several months [5]. Thus, there are unobserved transitions between states (uninfected and infected) that create a challenge for estimation of incidence and recovery rates. To address this challenge, we require specification of a class of continuous-time 2-state stochastic process models of infection status dynamics that must be shown to be consistent with observed data, and within which estimation of incidence and recovery rates is feasible.

The first attempt to carry out this program was by Bekessy et al. [6] using data from the Garki malaria surveys [3] in Kano State, Nigeria from 1970–1975. They introduced the 2-state continuous time Markov chains as candidates to represent the unobserved infection dynamics. The empirical question associated with this choice was whether or not a transition matrix *P*(Δ) *generated by a member of this class of models* could represent a transition matrix $\hat{P}\left(\text{Δ}\right)$ arising from field data. Here Δ is the time interval between observations collected at a survey date T_1_ and a later survey date T_2_. *P*(Δ) is a 2x2 transition matrix associated with a continuous time Markov chain with entries *p*_*i*,*j*_(Δ) = conditional probability that an individual has infection status *j* at the end of a time interval of length Δ given that his/her infection status at the beginning of the interval is *i*. Here, *i* and *j* can take on the values 1 = uninfected or 2 = infected. $\hat{P}\left(\text{Δ}\right)$ is a 2x2 stochastic matrix with entries *n*_*i*,*j*_/(*n*_*i*,1_ + *n*_*i*,2_), where *n*_*i*,*j*_ counts the number of individuals observed to be in state *i* at the beginning of interval Δ and in state *j* at the end of the interval. The entries are interpreted as conditional frequencies of observing a transition *i* → *j* between the consecutive survey dates.

For the Garki surveys, Δ ≈ 10 weeks. The transition matrices *P*(Δ) have a representation in terms of incidence and recovery rates given by:

$$\begin{array}{l} P\left(\text{Δ}\right)=\text{exp}\left(\text{Δ}Q\right)\\ Q=\left(\begin{array}{cc} -q_{1}{}^{} & q_{1}\\ q_{2}{}^{} & -q_{2}{}^{} \end{array}\right)\\ \text{with }q_{i}\geq 0\text{ for }i=1,2 \end{array}$$ (1)

Here *q*_1_ is the incidence rate at any time *t* in the interval Δ, and *q*_2_ is the corresponding recovery rate. Bekessy et al. [6] showed that a 2x2 stochastic matrix, P*, has a representation of the form Eq (1) if and only if

$$\text{trace }P^{*}\;>\;1$$ (2)

Thus, the empirically determined stochastic matrices $\hat{P}\left(\text{Δ}\right)$ can be generated by observations taken at two points in time on a continuous time Markov chain provided trace $\hat{P}\left(\text{Δ}\right)>1$. Formal statistical tests of this hypothesis were put forth by Singer & Cohen [7].

Interestingly, Bekessy et al. [6], Singer & Cohen [7], and Molineaux & Gramiccia [3] found that all pairs of consecutive surveys during the baseline period of data collection in the Garki project satisfied eq (2). However, there were pairs of consecutive surveys conducted during the intervention phase of the project where trace $\hat{P}\left(\text{Δ}\right)<1$. In these instances, Molineaux & Gramiccia [3] claimed that incidence and recovery rates were not estimable. While this assertion is correct for the class of model eq (1), the unanswered question as of 1980 was: what alternative and substantively defensible models could generate transition matrices satisfying trace $\hat{P}\left(\text{Δ}\right)<1$ and within which incidence and recovery rates could be estimated?

Surprisingly, to the best of our knowledge, this question has not been taken up in the past 40 years. Nevertheless, its importance stems from the fact that 2-wave panel data in a diversity of malaria surveys/surveillance projects have a majority of their transition matrices, $\hat{P}\left(\text{Δ}\right)$, satisfying trace $\hat{P}\left(\text{Δ}\right)<1$. In addition, incidence and recovery rates are important quantities for evaluation of malaria intervention programs.

The purposes of this paper are to: (i) present a class of models with associated 2x2 transition matrices, P*, within which those satisfying eq (1) are nested, some members of which satisfy trace P* < 1, and for all of which incidence and recovery rates can be calculated; (ii) exhibit identifiability and/or partial identifiability criteria arising from specific malaria contexts that ensure uniqueness, or highly constrained non-uniqueness of incidence and recovery rates; and (iii) apply the models and methods in (i) and (ii) to a time series of 2-wave panel data sets from Dar es Salaam, Tanzania as an example of the applicability of the proposed method [5,8,9]. To facilitate dissemination and utilization of the methodology by the malaria community, we developed a code to calculate incidence rates from longitudinal data utilizing the R package v.2.15.1 [10], which we make available as Supporting Information in a documented version (S1 Code) and as a R file (S2 Code). To allow replication of our results, we provide the Dar es Salaam data utilized in this paper (Tables 1 and 2).

**Table 1:** Elements of the 2x2 matrix and time interval between two consecutive survey rounds, extracted from the UMCP data, and used to estimate incidence rates by wards.

Consecutive pairs of survey rounds	Municipality	Ward	n11	n12	n21	n22	Time Interval
R12	Kinondoni	Magomeni	78	7	50	8	42.1
R12	Kinondoni	Mikocheni	76	12	13	1	40.2
R12	Kinondoni	Mwananyamala	35	23	2	5	28.3
R12	Kinondoni	Mzimuni	82	8	4	0	34.8
R12	Kinondoni	Ndugumbi	111	12	81	14	40.0
R12	Temeke	Azimio	28	48	1	8	29.1
R12	Temeke	Keko	64	2	2	0	32.8
R12	Temeke	Kurasini	115	20	46	7	41.6
R12	Temeke	Miburani	92	22	8	1	37.8
R12	Temeke	Mtoni	95	15	49	12	39.0
R12	Ilala	Buguruni	39	5	1	0	32.5
R12	Ilala	Ilala	104	5	47	2	40.1
R12	Ilala	Kipawa	88	18	59	6	42.0
R12	Ilala	Mchikichini	150	28	15	3	36.8
R12	Ilala	Vingunguti	32	27	2	2	29.1
R23	Kinondoni	Magomeni	146	26	23	4	41.8
R23	Kinondoni	Mikocheni	99	13	25	4	36.4
R23	Kinondoni	Mwananyamala	145	14	53	8	40.5
R23	Kinondoni	Mzimuni	138	18	23	5	40.6
R23	Kinondoni	Ndugumbi	142	6	29	1	39.0
R23	Temeke	Azimio	91	9	82	19	40.9
R23	Temeke	Keko	164	29	20	1	41.3
R23	Temeke	Kurasini	124	23	19	6	41.1
R23	Temeke	Miburani	121	23	18	2	39.5
R23	Temeke	Mtoni	111	12	29	1	39.0
R23	Ilala	Buguruni	148	22	32	4	42.2
R23	Ilala	Ilala	151	7	24	0	38.5
R23	Ilala	Kipawa	155	26	49	5	41.1
R23	Ilala	Mchikichini	167	7	33	6	38.5
R23	Ilala	Vingunguti	115	7	52	3	40.8
R34	Kinondoni	Magomeni	214	14	36	3	42.6
R34	Kinondoni	Mikocheni	171	7	22	0	44.2
R34	Kinondoni	Mwananyamala	197	13	42	2	45.9
R34	Kinondoni	Mzimuni	178	5	34	0	43.3
R34	Kinondoni	Ndugumbi	219	10	17	1	44.7
R34	Temeke	Azimio	99	18	27	3	45.3
R34	Temeke	Keko	137	7	33	0	43.5
R34	Temeke	Kurasini	212	8	41	1	43.5
R34	Temeke	Miburani	221	3	37	2	45.6
R34	Temeke	Mtoni	176	15	23	1	45.4
R34	Ilala	Buguruni	128	8	31	1	42.1
R34	Ilala	Ilala	161	2	11	0	45.6
R34	Ilala	Kipawa	210	9	40	1	42.5
R34	Ilala	Mchikichini	182	14	17	1	45.9
R34	Ilala	Vingunguti	125	14	10	2	45.5
R45	Kinondoni	Magomeni	186	3	15	0	36.2
R45	Kinondoni	Mikocheni	224	3	15	0	35.3
R45	Kinondoni	Mwananyamala	209	10	7	1	37.5
R45	Kinondoni	Mzimuni	201	1	8	0	39.1
R45	Kinondoni	Ndugumbi	154	3	15	0	36.1
R45	Temeke	Azimio	115	1	16	0	39.1
R45	Temeke	Keko	135	0	12	0	43.6
R45	Temeke	Kurasini	187	3	9	0	34.1
R45	Temeke	Miburani	92	3	3	0	38.1
R45	Temeke	Mtoni	110	10	16	1	36.5
R45	Ilala	Buguruni	178	27	7	1	38.8
R45	Ilala	Ilala	159	14	11	1	32.9
R45	Ilala	Kipawa	169	14	10	1	35.6
R45	Ilala	Mchikichini	139	11	12	1	33.7
R45	Ilala	Vingunguti	151	28	15	6	37.2
R56	Kinondoni	Magomeni	46	0	2	0	52.3
R56	Kinondoni	Mikocheni	143	2	2	0	50.8
R56	Kinondoni	Mwananyamala	87	0	6	0	51.1
R56	Kinondoni	Mzimuni	116	1	3	0	49.7
R56	Kinondoni	Ndugumbi	66	0	0	0	52.6
R56	Temeke	Azimio	67	1	0	0	51.1
R56	Temeke	Keko	104	1	3	0	48.9
R56	Temeke	Kurasini	63	0	1	0	49.1
R56	Temeke	Miburani	177	5	9	0	51.7
R56	Temeke	Mtoni	93	0	4	0	55.6
R56	Ilala	Buguruni	80	3	13	0	49.2
R56	Ilala	Ilala	50	2	5	0	46.3
R56	Ilala	Kipawa	146	7	12	0	56.5
R56	Ilala	Mchikichini	89	3	6	0	61.6
R56	Ilala	Vingunguti	106	2	6	0	50.7

Note: The column Time Interval (in weeks) represents the input **TimeInt** in the R code (S2 Code). The columns n11, n12, n21, and n22 are the elements of the 2x2 matrix described as **Data** in the R code.

**Table 2:** Elements of the 2x2 matrix and time interval (in weeks) between two consecutive survey rounds, extracted from the UMCP data, and used to estimate incidence rates by stage of the survey round.

Consecutive pairs of survey rounds	Stage	n11	n12	n21	n22	Time Interval
R12	I	95	98	5	15	28.9
R12	II	185	15	7	0	33.7
R12	III	318	62	36	5	37.9
R12	IV	310	32	177	28	39.7
R12	V	281	45	155	21	41.9
R23	I	351	30	187	30	40.7
R23	II	450	69	75	10	41.4
R23	III	387	43	76	12	38.2
R23	IV	404	25	82	2	38.8
R23	V	425	75	91	15	41.3
R34	I	421	45	79	7	45.6
R34	II	443	20	98	1	43.0
R34	III	574	24	76	3	45.3
R34	IV	556	27	51	2	45.2
R34	V	636	31	117	5	42.8
R45	I	475	39	38	7	37.8
R45	II	514	28	27	1	40.1
R45	III	455	17	30	1	35.3
R45	IV	423	27	42	2	35.0
R45	V	542	20	34	1	35.3
R56	I	260	3	12	0	51.0
R56	II	300	5	19	0	49.3
R56	III	409	10	17	0	54.1
R56	IV	209	2	9	0	52.5
R56	V	255	7	15	0	54.0

Note: The column Time Interval represents the input **TimeInt** in the R code (S2 Code). The columns n11, n12, n21, and n22 are the elements of the 2x2 matrix described as **Data** in the R code.

An important feature of our considerations is allowance for non-identifiability in situations where subject-matter-based constraints are too weak to ensure unique parameter identification from input information. Depending upon the particular setting, the extent of non-identifiability—or partial identifiability—may be sufficiently limited that the unidentified parameters are restricted to a narrow range of values, with accompanying incidence rates also varying over a small range. This is, indeed, the situation in Dar es Salaam, the site of our empirical data. As a consequence, we show how to explicitly incorporate variability due to a small degree of under-identification together with sampling variability to produce composite uncertainty intervals for incidence rates. Useful discussions of partial identifiability, including in the setting of mixture models, are given by Manski [11], Gustafson [12], and Henry et al. [13].

## Methods

### Ethics statement

Ethical approval to use the UMCP data was provided by the Harvard T.H. Chan School of Public Health Institutional Review Board (Protocol # 20323–101).

### Two-component mixtures of continuous time Markov chains

We begin with the observation that every 2x2 stochastic matrix, *P*, with non-zero entries can be a transition matrix for at least one 2-component mixture of continuous-time Markov chains. Thus, *P* has a representation of the form

P=SU+(I−S)V (3)

where *S* is a diagonal matrix with entries *s*_*i*_, i = 1,2 in the unit interval, [0,1]. *U* and *V* are each stochastic matrices having representations of the form eq (1). Hence they satisfy the condition eq (2): trace *U* > 1 and trace *V* > 1. It will be convenient to represent the matrices *P*, *U*, and *V* as points in the unit square; namely **p** = (*p*_1_,*p*_2_), **u** = (*u*_1_,*u*_2_), and **v** = (*v*_1_,*v*_2_). The coordinates *p*_*i*_,*u*_*i*_, and *v*_*i*_, *i* = 1,2 are the diagonal entries in *P*, *U*, and *V* respectively.

Depending on the empirical setting, we will also have occasion to consider data of the form **p** = (*p*_1_,0) and **p** = (0,*p*_2_); i.e. points on the boundary of the unit square. In the first of these conditions, *P* has a representation of the form eq (3), but with **u** = (1,0) (hence, trace *U* = 1) and trace *V* > 1. For **p** = (0,*p*_2_), the representation eq (3) also holds but now with **u** = (0,1) and trace *V* > 1. Data of the form **p** = (*p*_1_,0) occur frequently in the Dar es Salaam data, as discussed later. In these boundary cases, *U* still has a representation of the form eq (1) but with **q** = (0,−∞) when **p** = (*p*_1_,0), and **q** = (−∞,0) when **p** = (0,*p*_2_). In the first case, the interpretation of *q*_2_ = ∞ is that there is an infinitely fast recovery rate. This would be associated with a population where everyone is on prophylaxis, or where effective anti-malarial drugs are administered immediately following a diagnosis of infection. In the second case, *q*_1_ = ∞ corresponds to an infinitely fast incidence rate. This would be a situation where there is instantaneous new infection of any exposed individual.

For data corresponding to **p** in the interior of the unit square—i.e. $\hat{P}\left(\text{Δ}\right)$ with non-zero entries—we calculate the incidence and recovery rates for the mixture eq (3) via:

$$\begin{array}{l} r_{1}=s_{1}^{}q_{1}^{\left(U\right)}+\left(1-s_{1}\right)\;q_{1}^{\left(V\right)}\;\text{(incidence rate)}\\ r_{2}=s_{2}^{}q_{2}^{\left(U\right)}+\left(1-s_{2}\right)\;q_{2}^{\left(V\right)}\;(\text{recovery rate}) \end{array}$$ (4)

In terms of *U* and *V*, the rates $q_{i}^{\left(U\right)}$ and $q_{i}^{\left(V\right)}$, *i* = 1,2 can be expressed as (note that these formulas are entries in matrix logarithms of $U=e^{Q^{\left(U\right)}\text{Δ}}\;$ and $V=e^{Q^{\left(V\right)}\text{Δ}}\;$– see e.g. [7]):

$$\begin{array}{l} q_{i}^{\left(U\right)}=\frac{\text{log}\left(\text{trace }U-1\right)}{\text{trace }U-2}\frac{\left(1-u_{i}\right)}{\text{Δ}}\\ q_{i}^{\left(V\right)}=\frac{\text{log}\left(\text{trace }V-1\right)}{\text{trace }V-2}\frac{\left(1-v_{i}\right)}{\text{Δ}} \end{array}$$

When **p** = (*p*_1_,0), we have

$$\begin{array}{l} r_{1}=\left(1-s_{1}\right)\;q_{1}^{\left(V\right)}\\ \;=\left(1-s_{1}\right)\frac{\text{log}\left(\text{trace }V-1\right)}{\text{trace }V-2}\frac{\left(1-v_{1}\right)}{\text{Δ}}\\ \;=\frac{\text{log}\left(\text{trace }V-1\right)}{\text{trace }V-2}\frac{\left(1-p_{1}\right)}{\text{Δ}},\text{ since }s_{1}=\frac{p_{1}-v_{1}}{1-v_{1}} \end{array}$$ (5)

The recovery rate is ∞ for every *s*_2_ > 0. When *s*_2_ = 0, we have

$$r_{2}=\frac{\text{log}\left(\text{trace }V-1\right)}{\text{trace }V-2}\frac{\left(1-v_{2}\right)}{\text{Δ}}$$

An analogous argument yields *r*_1_ and *r*_2_ when **p** = (0,*p*_2_).

### Identification

We first re-express Eq (3) via the system of equations

$$\begin{array}{l} p_{1}=s_{1}^{}u_{1}^{}+\left(1-s_{1}\right)\;v_{1}^{}\\ p_{2}=s_{2}^{}u_{2}^{}+\left(1-s_{2}\right)\;v_{2}^{} \end{array}$$ (6)

where (*s*_*i*_,*u*_*i*_,*v*_*i*_) ∈ [0,1]^6^ for *i* = 1,2 and *u*_1_+*u*_2_>1, *v*_1_+*v*_2_>1.

Given **p** = (*p*_1_,*p*_2_), eq (6) is an under-identified system. Additional subject-matter motivated constraints must be imposed to either identify (**s,u,v**) uniquely or restrict this vector to a small set in [0,1]^6^∩((**u,v**):*u*_1_+*u*_2_>1, *v*_1_+*v*_2_>1). In the context of malaria in Dar es Salaam, we impose the constraints: *u*_1_ ≤ 0.2, *u*_2_ = 1, 0.9<*v*_1_ < 1, *v*_2_ < 0.5, *s*_1_ < 0.5, and *v*_1_ − *v*_2_ ‘large’.

A full rationale for the above restrictions will be given later when we present the Dar es Salaam case study. However, the central point here is that a system of constraints such as these is essential for parameter identification or partial identification. The conditions that *v*_1_ − *v*_2_ be ‘large’ and *v*_1_ < 1 require additional comment. First, it is a matter of judgment about what is a high probability of being observed uninfected at consecutive surveys of the *V*-process, while still not being a sure thing—i.e. *v*_1_ = 1. In identifying parameters, we first select *v*_1_ ∈[0.9,1) and secondarily choose *v*_2_ as small as possible consistent with the other constraints. Two examples will serve to illustrate the issues.

#### Example 1

Suppose **p** = (*p*_1_,*p*_2_) = (0.7,0.2). This is a case where trace *P* = *p*_1_ + *p*_2_ < 1. Now, set *v*_1_ = 0.95. Then (*s*_1_,*u*_1_) is a point on the curve 0.95*s*_1_ − *s*_1_*u*_1_ − 0.25 = 0, subject to the constraints *u*_1_ ≤ 0.2 and *s*_1_ < 0.5. This is a curve of admissibility for (*s*_1_,*u*_1_). Then, from 0.20 = *s*_2_ + (1 − *s*_2_)*v*_2_ and *v*_1_ + *v*_2_ > 1, we have *v*_2_ = [0.20 − *s*_2_]/(1 − *s*_2_) > 1 − *v*_1_ = 0.05. The left hand equality represents a curve of admissibility for (*s*_2_,*v*_2_). The two curves represent the extent of non-identifiability of the parameters in eq (6) given the a priori constraints for Dar es Salaam. This variation could be further reduced if, for example, we specified *s*_1_ based on additional empirical evidence.

Some judgment enters in choosing *v*_2_ > 0.05. We want *v*_2_ close to 0.05 to make *v*_1_ − *v*_2_ ‘large’, but not so close that *v*_1_ + *v*_2_ = 1 + *ε* yields an unreasonably small value of *ε*. The concern here is that the contribution to the overall incidence rate from the *V*-process is given by $q_{1}^{\left(V\right)}=\frac{\text{log}\varepsilon }{\varepsilon -1}\frac{\left(1-v_{1}\right)}{\text{Δ}}$, where Δ is the time between consecutive surveys. As *ε* → 0, $q_{1}^{\left(V\right)}\to \infty$. This is, of course, physically impossible. Thus, we choose *ε* = 0.008 based on the fact that larger values would not have much influence on $q_{1}^{\left(V\right)}$, while smaller values, by another power of 10 or more, would yield unrealistically high conversion rates from negative to positive parasitological status. Now, *ε* = 0.008 implies that *v*_2_ = 0.058 and *s*_2_ = 0.15. This leaves (*s*_1_,*u*_1_) on the curve 0.95*s*_1_ − *s*_1_*u*_1_ − 0.25 = 0 as the only source of non-identifiability. Table 3 shows the impact of this source of variation on the incidence rate *r*_1_.

**Table 3:** Influence of variation in (*s*_1_,*u*_1_)on the incidence rate *r*_1_.

*s*_1_	*u*_1_	*r*_1_
0.263	0.006	0.0381
0.300	0.117	0.0204
0.320	0.168	0.0184
0.330	0.192	0.0177
0.333	0.199	0.0175

In Table 3, $r_{1}=s_{1}^{}q_{1}^{\left(U\right)}+\left(1-s_{1}\right)\;q_{1}^{\left(V\right)}$, with $q_{i}^{\left(U\right)}=\frac{\text{log}\left(\text{trace }U-1\right)}{\text{trace }U-2}\frac{\left(1-u_{i}\right)}{\text{Δ}}$ and $q_{1}^{\left(V\right)}=\frac{\text{log}\varepsilon }{\varepsilon -1}\frac{\left(1-v_{1}\right)}{\text{Δ}}$. We set Δ = 40 weeks, which is roughly the average spacing between consecutive surveys in the Dar es Salaam data, as described later. The point (*s*_1_,*u*_1_) = (0.263, 0.006) corresponds to a very low probability of being observed uninfected at two consecutive surveys of the *U*-process. Thus, we retain the other 4 points as plausible values and include the variation in *r*_1_ corresponding to them in our overall assessment of variability in the incidence rate. We again emphasize that this is variability induced by non-identifiability. It is an important feature of the methodology introduced herein. Overall variation in incidence rates is a composite of sampling variability and non-identifiability variation, detailed later.

#### Example 2

Suppose **p** = (*p*_1_,0) with *p*_1_ > 0.5. With *v*_1_ + *v*_2_ = 1 + *ε*, if we set *ε* = 0.008 as in example 1, then *r*_1_ is given by eq (5) and the model is identified. The values of *v*_1_ and *v*_2_ are determined by the choice of *μ* > 0 in the specification *v*_1_ = (1 − *μ*)*p*_1_ with *μ* small. As a numerical example, suppose *p*_1_ = 0.9 and *μ* = 0.05. Then *v*_1_ = 0.95 x 0.9 = 0.855. Since 0.9 = *s*_1_ + (1 − *s*_1_)*v*_1_, we have *s*_1_ = 0.31. Then *v*_2_ = 1.008 − 0.855 = 0.153 and *v*_1_ − *v*_2_ = 0.702. Finally, we have $r_{1}=\left(1-s_{1}\right)\;q_{1}^{\left(V\right)}$, since $q_{i}^{\left(U\right)}=0$. Then *r*_1_ = 0.01217 using Δ = 40 weeks.

Although it does not arise in the Dar es Salaam example, we briefly indicate identification of **v** when **p** = (*p*_1_,0) and *p*_1_ < 0.5. Here we must have *v*_2_ > *v*_1_ as seen in the following simple, but generic, example. Suppose *p*_1_ = 0.4 and *v*_1_ = 0.95 *p*_1_ = 0.38. From 0.4 = *s*_1_ + (1 − *s*_1_)*v*_1_, we obtain *s*_1_ = 0.0322. Then from *v*_1_ + *v*_2_ = 1 + *ε*, we have *v*_2_ = 1 + *ε* − *v*_1_, and with *ε* = 0.008, *v*_2_ = 0.628. Then *v*_1_ − *v*_2_ = −0.248.

### Assessing variability of incidence rates

When **p** = (*p*_1_,*p*_2_) is in the interior of the unit square, we generate 1,000 tables by doing binomial sampling for row 1 with probability *p*_1_ and for row 2 with probability *p*_2_ with sample sizes *n*_11_ + *n*_12_ and *n*_21_ + *n*_22_, respectively. If, for a particular table, the system of eq (6) has a unique solution (**s,u,v**), subject to context-specific constraints, then we compute an incidence rate, *r*_1_, for that table. If there is a zone of non-identifiability, as previously exemplified by the equation 0.95*s*_1_ − *s*_1_*u*_1_ − 0.25 = 0 in Example 1, then we compute *r*_1_ for each of 100 values *u*_1_ (which then determines *s*_1_) subject to the a priori constraints on *s*_1_ and *u*_1_. This yields a set of incidence rates that reflect variation due to non-identification. We used the minimum, median, and maximum values of *r*_1_ from each such set of 100 values and viewed them as the summary rates for the particular table. Finally we take the summary rates, for tables where non-identifiability is an issue, and the unique rate for tables when the system eq (6) is identified, and rank this full set of rates. We designate the 2.5^th^ percentile and the 97.5^th^ percentile of the ranked list as the upper and lower bounds on a 95% variation interval for the incidence rate of the original table. This takes both sampling variability and variation due to non-identifiability into account.

When **p** = (*p*_1_,0), we treat the 0 as a structural zero—in the case of Dar es Salaam—and only do binomial sampling on the first row to generate 1,000 tables having this same structure. We then describe the variation in *r*_1_ in the same manner as indicated above. In applications where **p** = (*p*_1_,0) does not have a structural zero, we perturb the second coordinate to a small value—e.g. 10^−5^ or less—and do binomial sampling for the second row with this value. Then we proceed as in the above paragraph to calculate a confidence interval for *r*_1_.

### Submicroscopic infection

Light microscopy has limitations as a technology for diagnosing *Plasmodium* *falciparum* infections, particularly in low transmission settings [14–16]. In a recent study of Okell et al. [16], the supplementary information for the paper contains an especially interesting and useful table comparing prevalence estimates using microscopy and PCR on the same blood samples. The data come from a wide variety of settings, and exhibit considerable variation in prevalence rates as ascertained via microscopy. The prevalence ratio, *p =* [prevalence rate from microscopy]/[prevalence rate from PCR] provides a basis for adjusting empirical microscopy rates to what you would expect to find if PCR had actually been done on the same blood samples. This calculation will, of course, only yield adjusted prevalence rates. For our longitudinal data, it would have been desirable to have microscopy and PCR based estimates of *p*_12_,*p*_21_ and *p*_22_, from which we could directly recover *p*_11_. However, the lack of identifiability of transition probabilities from prevalence rates can still be dealt with in particular settings, such as Dar es Salaam, by invoking an additional, and obviously context dependent, constraint. We exhibit the methodology on **p** = (0.7, 0.2)–the transition rates in example 1–augmented by a table of counts with entries *n*_*ij*_ consistent with these values. Using an adjusted table of counts, $n_{ij}^{*}$, and thereby an adjusted vector, $p^{*}=\left(p_{1}^{*},p_{2}^{*}\right)$, we calculate the incidence rate, $r_{1}^{*}$, that represents what we might have expected to find if PCR had been done on the blood samples in Dar es Salaam.

We introduce the table of counts {*n*_*ij*_,1 ≤ *i*, *j* ≤ 2} with *n*_11_ = 100, *n*_12_ = 43, *n*_21_ = 90, and *n*_22_ = 23. For this table, **p** = (0.7, 0.2). It is one of a myriad of tables that could have been selected to illustrate our points about submicroscopic infection. However, it is comparable in size to many of the sub-ward tables in the Dar es Salaam data, and thus especially useful for illustrating an adjustment methodology.

The prevalences at the initial and final rounds of data collection for the above table are: at initial survey = [90 + 23]/256 = 0.4414, and at final survey = [23 + 43]/256 = 0.2578. From Table S1 in Okell et al. [16], we find the prevalence from microscopy that is closest to the prevalence at initial survey in Dar es Salaam given by 0.4414. This is the prevalence of 0.481 based on data from Guinea Bissau. The corresponding prevalence ratio is *p =* 0.551. Thus our estimate for a corresponding PCR-based prevalence rate at the initial survey is 0.4414/0.551 = 0.8011. In contrast to the initial survey situation, there are four nearby microscopy-based prevalence rates to associate with the prevalence rate for the final survey given above by 0.2578. These values, their associated prevalence ratios, and our estimate for the corresponding PCR-based prevalence rates are shown in Table 4. Each PCR rate is equal to 0.2578/*p*.

**Table 4:** Microscopy and PCR-based prevalence rates and prevalence ratios.

Microscopy rate	Prevalence ratio, *p*	PCR rate
0.246	0.565	0.456
0.247	0.521	0.495
0.250	0.891	0.289
0.255	0.331	0.829

Source: Table S1 of Okell et al. [36].

For our analysis, we use the average of these PCR rates, namely 0.5173. To obtain an associated table of counts $n_{ij}^{*}$, we first observe that $n_{11}^{*}+n_{12}^{*}+n_{21}^{*}+n_{22}^{*}=256=$ total count from the microscopy-based table with entries *n*_*ij*_. From PCR prevalence at initial survey = 0.8011, we obtain $n_{21}^{*}+n_{22}^{*}=205$. From PCR prevalence at final survey = 0.5173, we obtain $n_{12}^{*}+n_{22}^{*}=132$. Adding and subtracting $n_{22}^{*}$ to the equation for total count, we can rewrite it as $\left(n_{11}^{*}-n_{22}^{*}\right)+n_{12}^{*}+n_{22}^{*}+n_{21}^{*}+n_{22}^{*}=256$. Then we have that $n_{11}^{*}-n_{22}^{*}=-81$. To be consistent with the microscopy-based vector, **p** = (0.7, 0.2), where obviously *p*_1_ > *p*_2_, we choose $n_{11}^{*}$ to ensure that $p_{1}^{*}>p_{2}^{*}$. Table 5 shows some choices for estimated PCR-based tables.

**Table 5:** An example of incidence rates, $r_{1}^{*}$, along the curve of $\left(s_{1}^{*},u_{1}^{*}\right)$ values based on PCR-based tables.

$n_{11}^{*}$	$n_{22}^{*}$	$p_{1}^{*}$	$p_{2}^{*}$	$n_{12}^{*}$	$n_{21}^{*}$	$s_{1}^{*}$	$u_{1}^{*}$	$q_{1}^{\left(U\right)*}$	$r_{1}^{*}$
10	91	0.244	0.444	41	114	0.1758	0.006	0.1279	0.0244
30	111	0.588	0.541	21	94	0.1953	0.100	0.0576	0.0132
40	121	0.784	0.590	11	84	0.2210	0.199	0.0404	0.0107

Here we use as an example $p_{1}^{*}=0.784$ and $p_{2}^{*}=0.590$ (third line of Table 5) for subsequent calculations. To obtain what is interpreted as a PCR-based incidence rate, we proceed as in the previously described microscopy-based rate calculations. First set $v_{1}^{*}=0.95$. Then from $p_{1}^{*}=s_{1}^{*}u_{1}^{*}+\left(1-s_{1}^{*}\right)\;v_{1}^{*}$, we obtain $0.95s_{1}^{*}-s_{1}^{*}u_{1}^{*}-0.166=0$. Using $v_{2}^{*}=\left[0.590-\;s_{2}^{*}\right]/\left(1-s_{2}^{*}\right)>1-v_{1}^{*}=0.05$, we set $s_{2}^{*}=0.5$. Then $v_{2}^{*}=0.18$, and $v_{1}^{*}+v_{2}^{*}=1+\varepsilon =1.13$. Thus $q_{1}^{\left(V\right)*}=\frac{\text{log}\varepsilon }{\varepsilon -1}\frac{\left(1-v_{1}^{*}\right)}{\text{Δ}}=0.00173$, with *ε* = 0.13 and Δ = 40. Along the curve of $\left(s_{1}^{*},u_{1}^{*}\right)$ values given by $0.95s_{1}^{*}-s_{1}^{*}u_{1}^{*}-0.166=0$, we obtain values of $q_{1}^{\left(U\right)*}$ and $r_{1}^{*}$ as indicated in Table 5.

## Results

### Case study: Malaria in Dar es Salaam

#### Dar es Salaam

Dar es Salaam is the largest city and economic capital of the United Republic of Tanzania with a population of 4.4 million as recorded by the 2012 Population Census. The city is divided into three municipalities: (i) Ilala, the administrative area, where almost all government Offices and ministries are located; (ii) Temeke, the industrial area, which holds the main manufacturing centers and the Dar es Salaam Port, and has the largest concentration of low income dwellers; and (iii) Kinondoni, the most populated municipality that concentrates most of the high income housing in the city. Municipalities are divided into wards, and the smallest administrative component is the ten-cell unit.

Analogous to other urban areas in sub-Saharan Africa, fast urban growth was observed in Dar es Salaam in recent decades, but not accompanied by effective provision of infrastructure. It is estimated that in Dar es Salaam 70% of the population lives in unplanned settlements [17], with precarious access to improved drinking water, sanitation [18], and waste collection. Although an extensive network of drains is available [19–21], many of the drains lack proper and regular maintenance resulting in waste accumulation, flooding, water stagnation, and the proliferation of breeding sites for disease vectors [22,23].

The dominant malaria vectors in Dar es Salaam are *Anopheles gambiae s*.*s*. and *An*. *funestus* [24], and they breed mostly in aquatic habitats found in drains, ditches, construction pits, other human-made roles, and habitats associated with urban agriculture [23]. *Plasmodium falciparum* is responsible for more than 90% of malaria infections, and transmission is perennial [25] with seasonal variations related to the rainy seasons.

#### Dar es Salaam Urban Malaria Control Program (UMCP)

The methodology here proposed was applied to data from the UMCP, a large-scale larviciding intervention conducted in urban Dar es Salaam [8]. Reasons to choose the UMCP were three-fold. First, the availability of longitudinal data, specifically time series of two-wave panel data sets; six survey rounds were collected over a period of 4 years and 7 months (May-2004 to Dec-2008). Second, the intervention was shown to be successful [5,24,26], with the prevalence of malaria infection decreasing from 20.8% (95% CI: 16.8–24.9%) in the first survey round to 1.7% (95% CI: 1.4–2.1%) in the last [5]. Thus, the data describe a scenario of declining transmission to very low levels, similar to what is currently observed in countries/regions reaching malaria pre-elimination stages. Third, the calculation of incidence rates was a necessary input for the cost-effectiveness analysis of interventions [9].

**Fig 1:**
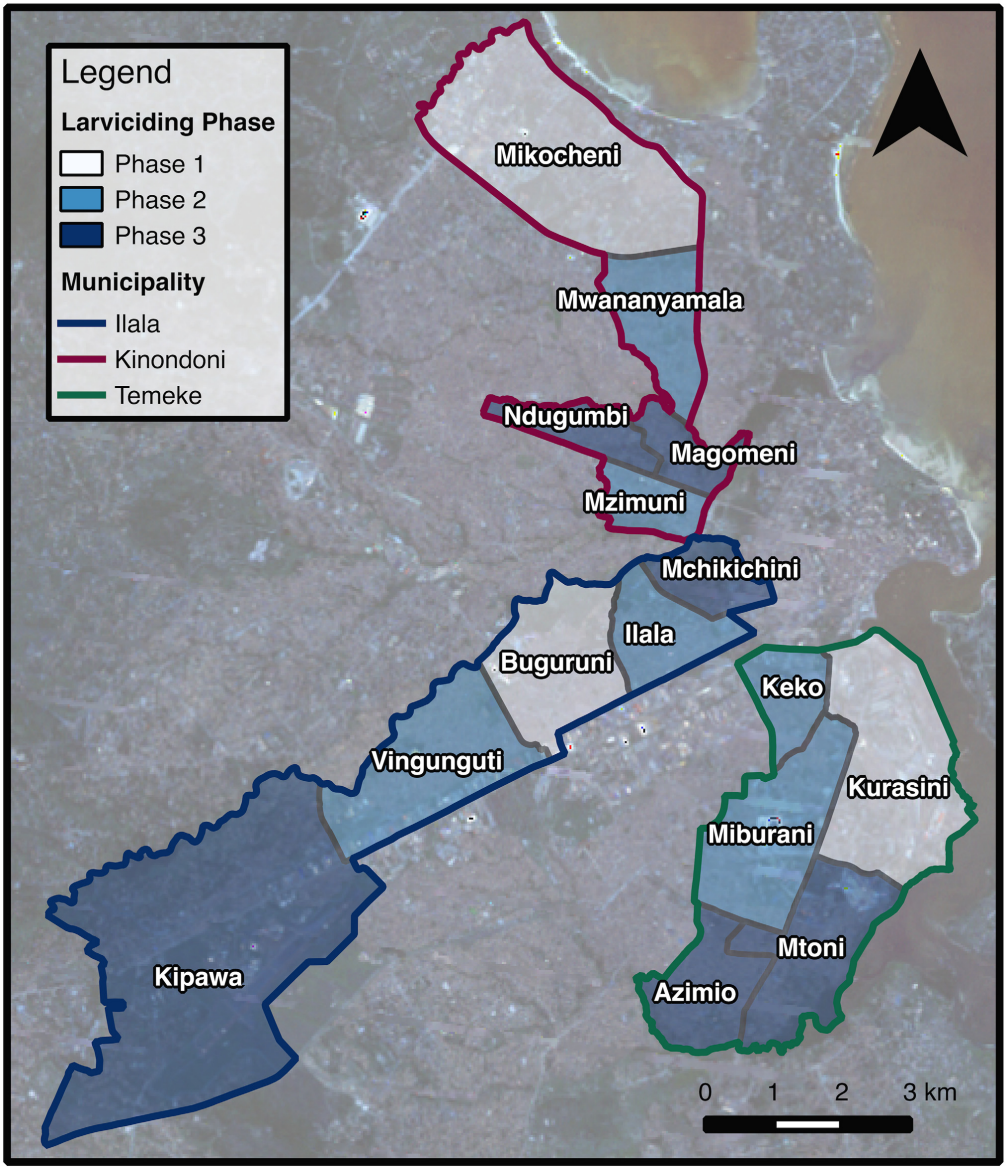
Urban Malaria Control Program wards, Dar es Salaam. The UMCP covered 15 of the 73 wards in Dar es Salaam. Larviciding was implemented in three phases such that the coverage moved from three wards to nine, and then to all 15 between 2006 and 2008.

**Fig 2:**
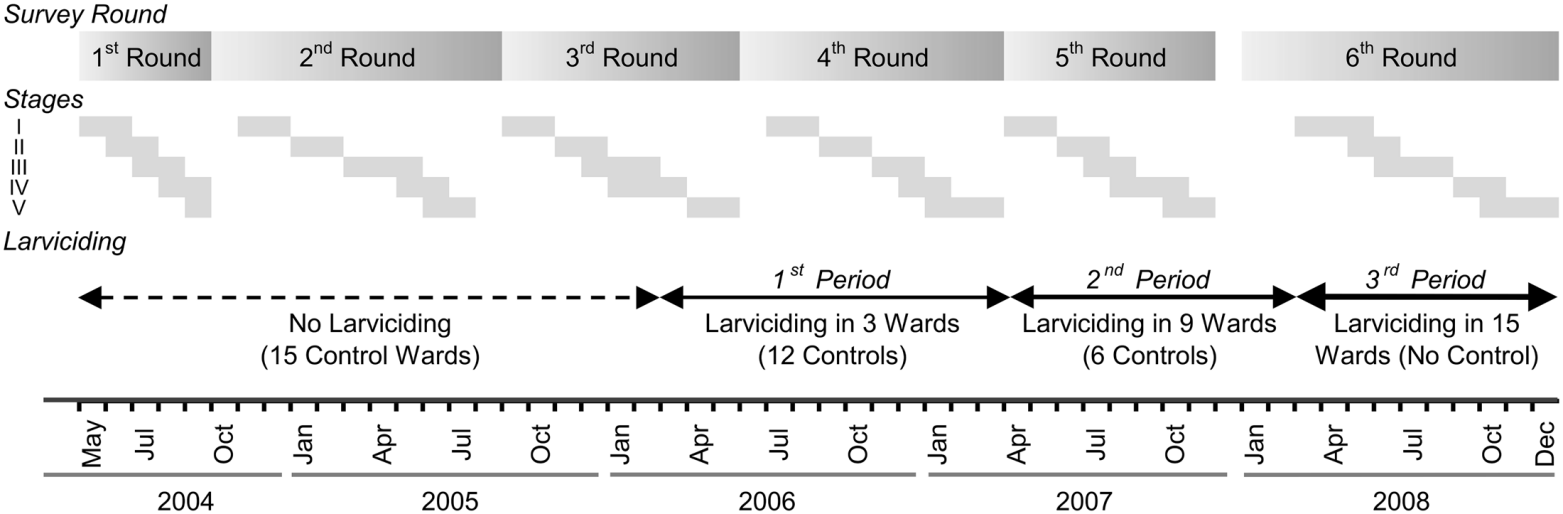
Timeline of data collection and larviciding intervention. The first survey round was launched in 05/2004. The first phase of the larviciding intervention started on 03/2006, the second on 05/2007, and the last on 04/2008. Each survey round had five stages, and three wards of each municipality are included in each stage. Month overlaps reflect slightly different duration of data collection in each ward. The wards included in each stage were: Stage I—Vingunguti, Mwananyamala, and Azimio; Stage II—Buguruni, Mzimuni, and Keko; Stage III—Mchikichini, Mikocheni, and Miburani; Stage IV—Ilala, Ndugumbi, and Mtoni; and Stage V—Kipawa, Magomeni, and Kurasini.

The UMCP started in 2004 targeting 15 wards (Fig 1), five in each of the three municipalities, covering approximately 56 km^2^ and more than 610,000 habitants [8]. After a period of baseline data collection and local capacity building, the program introduced the use of larvicides in three phases: in March 2006 it was introduced in 3 wards; in April 2007 it was expanded to 6 additional wards; and in April 2008 all 15 wards were being treated with larvicides. The time period of the survey rounds did not exactly match the launching of the intervention, so wards were exposed to larval control for different periods of time (Fig 2).

The UMCP wards revealed some variability regarding behavioral, socioeconomic, and geographical characteristics. While individual knowledge about malaria transmission was similar across wards, an index of household assets and, especially, housing conditions (quality of walls, screening, and source of water) showed important differences. For example, 47% of houses interviewed in Ilala had access to a good source of water, while in Temeke this number was only 19%. Use of a mosquito net, however, was relatively high, but declined significantly following the successful implementation of larval control [27]. The organization of the urban space was very heterogeneous, combining patches of planned areas (both higher and lower housing quality), squatter dwellings, urban agriculture, open spaces, industry, commerce, and areas of recent expansion (where foundations of houses, usually unfinished, often accumulate rainwater and offer conditions for mosquito breeding).

#### Data structure

Data available for estimating incidence rates consist of a time series of 2-wave panels covering six survey rounds collected over a period of 4 years and 7 months (May-2004 to Dec-2008), in the 15 UMCP wards [5,8,24]. Each survey round had five stages, and in each stage interviews were conducted in 3 out of the 15 UMCP wards (one in each municipality)–the order of the municipalities interviewed in each stage remained the same throughout the study (Fig 2). Therefore, five 2-wave panel data sets are available including baseline and intervention phases. A total of 5,223 participants were followed up twice, 2,349 three times, 1,236 four times, 472 five times, and 99 individuals participated in every survey round. Intervals between consecutive rounds of data collection varied from 6–12 months. Thus, repeated observations on the same individual can be separated by one or more unobserved changes in infection status (interval-censored data).

Individuals in the survey were tested for a malaria infection, upon consent. Finger-pricked blood samples were analyzed using Giemsa-stained thick smear microscopy. Quality check was conducted on a 10% sample of blood slides at the Muhimbili University of Health and Allied Sciences—MUHAS (a center of excellence in laboratory analysis), indicating a 94.5% specificity rate and 95.7% sensitivity rate [22]. All individuals found to be infected with malaria were treated with appropriate front-line regimens—sulphadoxine-pyrimethamine until August 2006, after which it was replaced with artesunate-amodiaquine.

#### Rationale for model constraints

We assume that there are two types of individuals governed, respectively, by the Markov chains with transition probabilities *U* and *V*. When **p** ∈ (0,1)^2^ = interior of the unit square, *U* represents people who do not follow anti-malarial drug regimens if they become infected, who do not sleep under bed nets, and whose houses lack adequate screening and tend to be in close proximity to larval habitats. Once infected, they remain so—also experiencing some superinfection—for sustained periods of time. This is reflected in the a priori assumptions that *u*_1_ ≤ 0.2 and *u*_2_ = 1.

The *V* process represents individuals who go for a diagnosis at the first sign of possible malaria symptoms and follow a prescribed drug treatment regimen if they are found to be infected. The quantification of this behavior is to have *v*_2_ small (and certainly < 0.5), which implies a high frequency, 1 − *v*_2_, of infected to uninfected transitions between surveys. Since all infected individuals are treated, a large value of 1 − *v*_2_ implies that the treatment regimen is usually followed. Further, reinfection rates will be low (corresponding to high *v*_1_, and hence low 1 − *v*_1_). The *V*-process people usually sleep under bednets, live in houses with effective screening of doors and windows, and generally, do not expose themselves to the local anopheles mosquitoes. This leads to the requirement that *v*_1_ − *v*_2_ should be large.

Finally, we assume that the *U*-process people are a minority in a locality where a control program is operative and local knowledge about malaria tends to be high. Thus, we assume, as a minimal condition, that *s*_1_ < 0.5.

When **p** = (*p*_1_,0), we assume that *p*_2_ = 0 is a structural zero. Thus, anyone found infected is transferred to the uninfected state via a regimen of anti-malarial drugs. Then *U* takes on the special form **u** = (1,0), and $q_{1}^{\left(U\right)}=0$, $q_{2}^{\left(U\right)}=+\infty$. This means that there are no transitions from uninfected to infected, and immediate transition from infected to uninfected. The *V* population operates under the same assumptions discussed above.

#### Estimated incidence rates in the context of the UMCP (based on microscopy)

**Fig 3:**
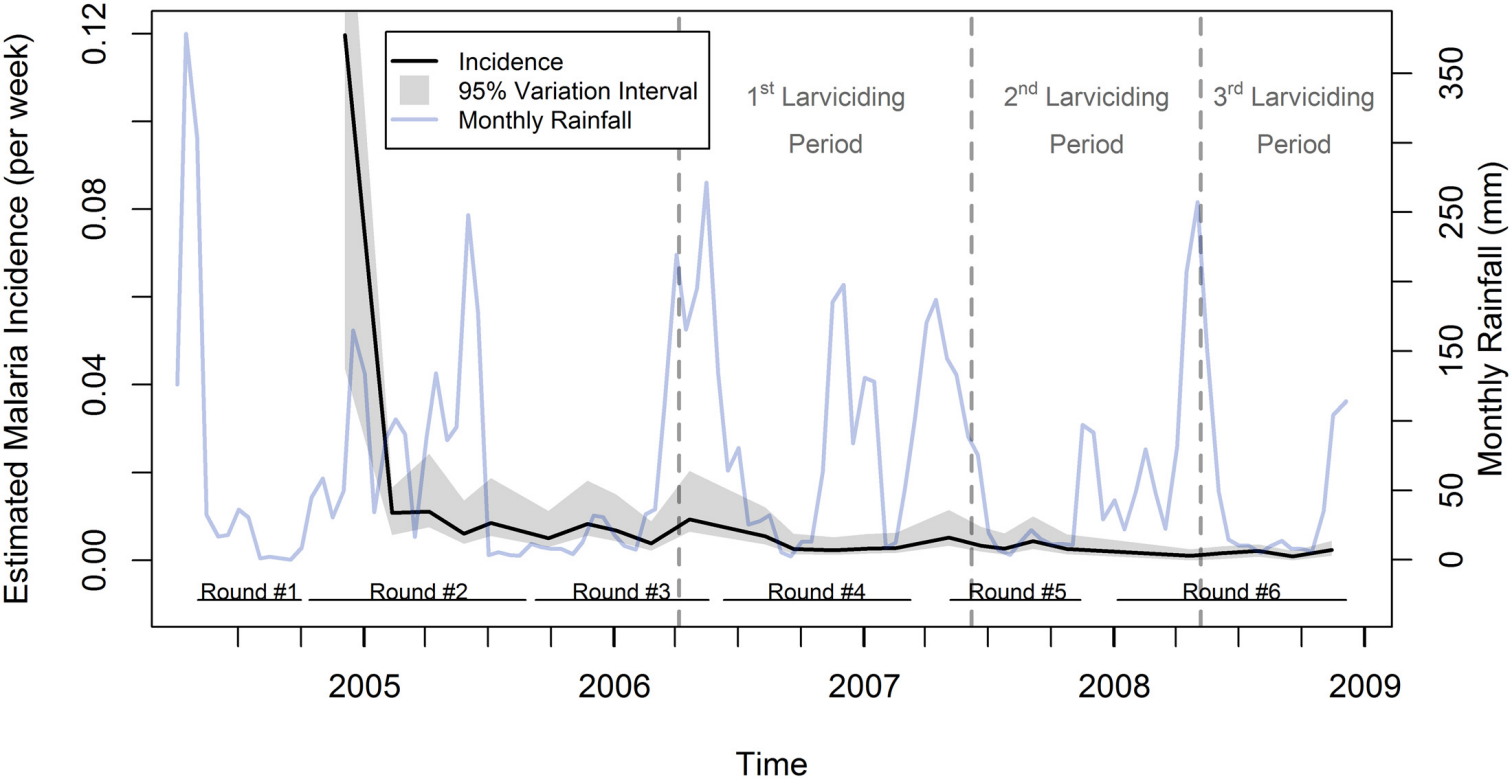
Estimated incidence rates, variability intervals, and 1-month lagged rainfall by stages of the survey rounds. Variability intervals consider the uncertainty due to sampling and partial identifiability of the mixture model. For ease of visualization, the variability interval for the first point in the graph was truncated. Each survey round of the UMCP conducted interviews in three wards at a time, or stages. Thus, each round had five stages in order to cover all 15 wards. Rainfall estimates were obtained from the National Oceanic and Atmospheric (NOAA) Climate Prediction Center (CPC), have a spatial resolution of eight kilometers [35], and were lagged by 30 days.

**Fig 4:**
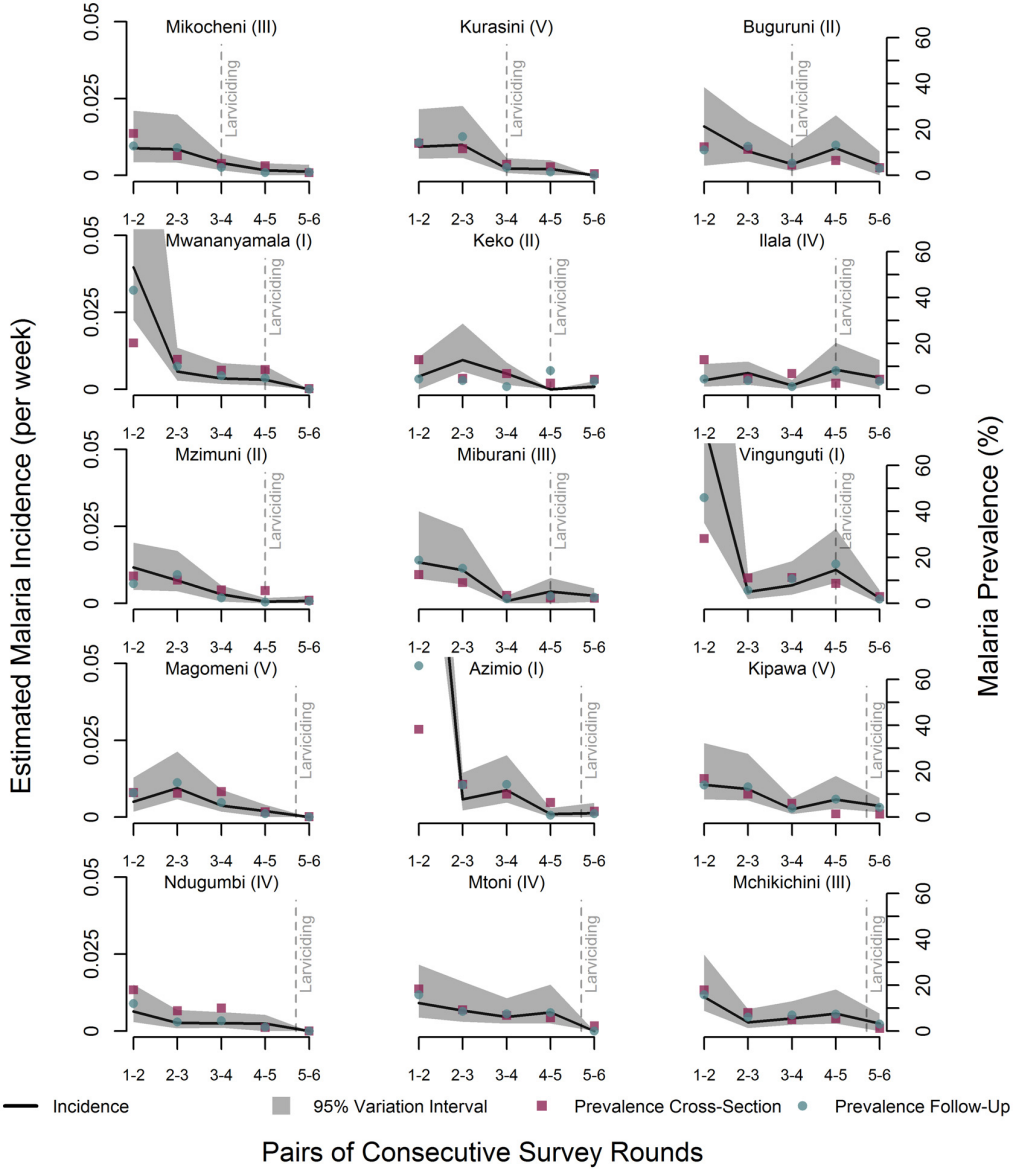
Estimated incidence rates, variability intervals, and prevalence of malaria infection by wards and consecutive pairs of survey rounds. Variability intervals consider the uncertainty due to sampling and partial identifiability of the mixture model. Prevalence rates were calculated using two distinct sets of data. First, the longitudinal data also utilized for the calculation of incidence rates (Prevalence—Follow-Up). Second, cross-sectional data collected at the same time and wards of the longitudinal data, but in different tencell units (Prevalence—Cross-Section). Each column of graphs shows wards from one municipality: the first column has wards from Kinondoni, the second column those from Temeke, and the third column shows wards from Ilala. Wards in the first line of the Figure were included in the first phase of the intervention; second and third line of graphs include wards targeted during the second phase of the intervention; and the last two lines show the wards included in the third and last phase of the intervention. The line indicating the onset of the larval control was placed on the pair of survey round at which any impact of each phase of the larval control could be observed. The Roman number after the name of the ward indicates the stage when interviews were conducted in each survey round (refer to Fig 2). For ease of visualization, estimates and variability intervals for Azimio and Vingunguti in R12 were truncated, as well as the variability interval for Mwananyamala in R12.

Estimates of incidence rates (cases per week) based on the UMCP data, detailed by pairs of consecutive survey rounds (Fig 3 and Table 6) and by ward (Fig 4 and Table 7) were obtained utilizing the methodology here proposed. Estimated rates ranged from 0.1202 cases per week in R12 (0.1537 in Azimio ward)–baseline period when no larviciding had been launched yet—to 0.0010 cases per week in R56 (a third of the wards had a rate of zero)–the intervention achieved full coverage in R6. An overall declining trend was observed, even before larviciding was introduced in three wards in March 2006 [5]. As the coverage of the intervention expanded, fluctuation in the rates was reduced, and rather stable rates were observed during R56. Just for illustration, we also plotted rainfall information lagged by one month [5] in Fig 3. Although seasonality in malaria transmission following the rains is often observed, rainfall patterns per se cannot fully explain fluctuations in incidence and prevalence rates, since many other factors can potentially overcome or augment the impact of rains [5].

**Table 6:** Estimated incidence rates and variability intervals by stages of the survey rounds.

Pairs of survey rounds	Stage	Estimated incidence rate	Variability interval due to partial identifiability		Variability interval considering partial identifiability and sampling variability					
			2.5^th^	97.5^th^	2.5^th^	97.5^th^	2.5^th^ of Minima	97.5^th^ of Maxima	Minima	Maxima
R12	I	0.12489	0.11395	0.16314	0.04345	0.16462	0.03847	0.18411	0.02349	0.18935
R12	II	0.01084	0.01084	0.01084	0.00578	0.01662	0.00578	0.01662	0.00289	0.02096
R12	III	0.01102	0.00875	0.02296	0.00756	0.02430	0.00657	0.05292	0.00531	0.05635
R12	IV	0.00603	0.00479	0.01257	0.00379	0.01363	0.00326	0.03135	0.00252	0.03791
R12	V	0.00844	0.00670	0.01760	0.00556	0.01873	0.00487	0.04135	0.00398	0.04788
R23	I	0.00495	0.00393	0.01032	0.00308	0.01127	0.00260	0.02554	0.00195	0.02873
R23	II	0.00823	0.00653	0.01715	0.00567	0.01819	0.00506	0.03873	0.00394	0.04657
R23	III	0.00670	0.00532	0.01397	0.00435	0.01502	0.00380	0.03314	0.00245	0.03917
R23	IV	0.00384	0.00305	0.00801	0.00226	0.00887	0.00194	0.02022	0.00133	0.02617
R23	V	0.00929	0.00738	0.01937	0.00653	0.02041	0.00585	0.04360	0.00526	0.05127
R34	I	0.00542	0.00430	0.01130	0.00364	0.01209	0.00322	0.02656	0.00246	0.03122
R34	II	0.00257	0.00204	0.00536	0.00142	0.00611	0.00121	0.01394	0.00061	0.01790
R34	III	0.00227	0.00180	0.00473	0.00130	0.00527	0.00112	0.01206	0.00067	0.01389
R34	IV	0.00263	0.00209	0.00547	0.00155	0.00602	0.00138	0.01354	0.00107	0.01655
R34	V	0.00278	0.00221	0.00579	0.00171	0.00630	0.00148	0.01456	0.00106	0.01733
R45	I	0.00514	0.00408	0.01072	0.00336	0.01152	0.00290	0.02601	0.00239	0.03315
R45	II	0.00330	0.00262	0.00687	0.00204	0.00761	0.00185	0.01731	0.00139	0.02004
R45	III	0.00261	0.00208	0.00545	0.00138	0.00614	0.00121	0.01369	0.00060	0.01724
R45	IV	0.00439	0.00348	0.00914	0.00264	0.01003	0.00230	0.02325	0.00179	0.02827
R45	V	0.00258	0.00205	0.00538	0.00143	0.00594	0.00132	0.01347	0.00061	0.01747
R56	I	0.00109	0.00109	0.00109	0.00000	0.00254	0.00000	0.00254	0.00000	0.00363
R56	II	0.00162	0.00162	0.00162	0.00032	0.00324	0.00032	0.00324	0.00000	0.00453
R56	III	0.00215	0.00215	0.00215	0.00086	0.00365	0.00086	0.00365	0.00043	0.00451
R56	IV	0.00088	0.00088	0.00088	0.00000	0.00220	0.00000	0.00220	0.00000	0.00352
R56	V	0.00241	0.00241	0.00241	0.00103	0.00447	0.00103	0.00447	0.00034	0.00550

**Table 7:** Estimated incidence rates and variability intervals by wards and consecutive pairs of survey rounds.

Pairs of survey rounds	Ward	Estimated incidence rates	Variability interval due to partial identifiability		Partial Identifiability & Sampling Variability					
			2.5^th^	97.5^th^	2.5^th^	97.5^th^	2.5^th^ of Minima	97.5^th^ of Maxima	Minima	Maxima
**Municipality: Ilala**										
R12	Buguruni	0.0170	0.0170	0.0170	0.0034	0.0306	0.0034	0.0306	0.0000	0.0442
R12	Ilala	0.0029	0.0023	0.0061	0.0007	0.0084	0.0005	0.0227	0.0000	0.0295
R12	Kipawa	0.0104	0.0082	0.0216	0.0060	0.0243	0.0050	0.0579	0.0027	0.0690
R12	Mchikichini	0.0109	0.0087	0.0228	0.0068	0.0246	0.0058	0.0559	0.0040	0.0665
R12	Vingunguti	0.0519	0.0428	0.0970	0.0263	0.1565	0.0189	0.1827	0.0117	0.2025
R23	Buguruni	0.0078	0.0062	0.0164	0.0046	0.0182	0.0039	0.0414	0.0011	0.0538
R23	Ilala	0.0056	0.0056	0.0056	0.0016	0.0104	0.0016	0.0104	0.0000	0.0128
R23	Kipawa	0.0090	0.0071	0.0187	0.0054	0.0208	0.0046	0.0480	0.0038	0.0546
R23	Mchikichini	0.0027	0.0021	0.0056	0.0009	0.0071	0.0006	0.0192	0.0003	0.0237
R23	Vingunguti	0.0036	0.0029	0.0075	0.0013	0.0093	0.0012	0.0239	0.0000	0.0298
R34	Buguruni	0.0036	0.0028	0.0075	0.0014	0.0092	0.0014	0.0225	0.0000	0.0294
R34	Ilala	0.0013	0.0013	0.0013	0.0000	0.0033	0.0000	0.0033	0.0000	0.0059
R34	Kipawa	0.0025	0.0020	0.0052	0.0009	0.0063	0.0009	0.0149	0.0004	0.0181
R34	Mchikichini	0.0040	0.0032	0.0083	0.0020	0.0097	0.0018	0.0231	0.0011	0.0286
R34	Vingunguti	0.0057	0.0045	0.0118	0.0029	0.0137	0.0025	0.0329	0.0010	0.0423
R45	Buguruni	0.0087	0.0069	0.0181	0.0053	0.0196	0.0048	0.0436	0.0035	0.0523
R45	Ilala	0.0063	0.0050	0.0131	0.0032	0.0154	0.0028	0.0365	0.0014	0.0470
R45	Kipawa	0.0055	0.0044	0.0115	0.0028	0.0133	0.0025	0.0304	0.0015	0.0396
R45	Mchikichini	0.0056	0.0044	0.0116	0.0023	0.0137	0.0020	0.0334	0.0004	0.0432
R45	Vingunguti	0.0108	0.0085	0.0224	0.0067	0.0246	0.0057	0.0565	0.0039	0.0639
R56	Buguruni	0.0036	0.0036	0.0036	0.0000	0.0072	0.0000	0.0072	0.0000	0.0119
R56	Ilala	0.0040	0.0040	0.0040	0.0000	0.0101	0.0000	0.0101	0.0000	0.0162
R56	Kipawa	0.0039	0.0039	0.0039	0.0011	0.0068	0.0011	0.0068	0.0000	0.0096
R56	Mchikichini	0.0026	0.0026	0.0026	0.0000	0.0060	0.0000	0.0060	0.0000	0.0077
R56	Vingunguti	0.0018	0.0018	0.0018	0.0000	0.0044	0.0000	0.0044	0.0000	0.0062
**Municipality: Kinondoni**										
R12	Magomeni	0.0050	0.0040	0.0104	0.0018	0.0129	0.0017	0.0332	0.0006	0.0415
R12	Mikocheni	0.0087	0.0069	0.0181	0.0042	0.0210	0.0040	0.0503	0.0011	0.0615
R12	Mwananyamala	0.0359	0.0285	0.0748	0.0225	0.1382	0.0160	0.1808	0.0106	0.1956
R12	Mzimuni	0.0124	0.0124	0.0124	0.0047	0.0202	0.0047	0.0202	0.0000	0.0279
R12	Ndugumbi	0.0062	0.0050	0.0130	0.0029	0.0150	0.0025	0.0382	0.0012	0.0463
R23	Magomeni	0.0093	0.0073	0.0193	0.0056	0.0213	0.0048	0.0482	0.0031	0.0592
R23	Mikocheni	0.0082	0.0065	0.0170	0.0041	0.0198	0.0035	0.0486	0.0015	0.0583
R23	Mwananyamala	0.0056	0.0044	0.0116	0.0028	0.0135	0.0022	0.0323	0.0016	0.0416
R23	Mzimuni	0.0073	0.0058	0.0152	0.0040	0.0173	0.0032	0.0407	0.0025	0.0485
R23	Ndugumbi	0.0027	0.0021	0.0056	0.0008	0.0073	0.0007	0.0189	0.0000	0.0275
R34	Magomeni	0.0037	0.0029	0.0077	0.0018	0.0090	0.0015	0.0214	0.0006	0.0296
R34	Mikocheni	0.0043	0.0043	0.0043	0.0012	0.0080	0.0012	0.0080	0.0000	0.0099
R34	Mwananyamala	0.0035	0.0027	0.0072	0.0017	0.0084	0.0015	0.0206	0.0004	0.0267
R34	Mzimuni	0.0031	0.0031	0.0031	0.0006	0.0061	0.0006	0.0061	0.0000	0.0080
R34	Ndugumbi	0.0025	0.0020	0.0052	0.0011	0.0065	0.0010	0.0155	0.0004	0.0232
R45	Magomeni	0.0021	0.0021	0.0021	0.0000	0.0050	0.0000	0.0050	0.0000	0.0071
R45	Mikocheni	0.0018	0.0018	0.0018	0.0000	0.0043	0.0000	0.0043	0.0000	0.0055
R45	Mwananyamala	0.0031	0.0025	0.0065	0.0014	0.0076	0.0012	0.0181	0.0005	0.0265
R45	Mzimuni	0.0006	0.0006	0.0006	0.0000	0.0025	0.0000	0.0025	0.0000	0.0043
R45	Ndugumbi	0.0026	0.0026	0.0026	0.0000	0.0060	0.0000	0.0060	0.0000	0.0077
R56	Magomeni	0.0000	0.0000	0.0000	0.0000	0.0000	0.0000	0.0000	0.0000	0.0000
R56	Mikocheni	0.0013	0.0013	0.0013	0.0000	0.0033	0.0000	0.0033	0.0000	0.0053
R56	Mwananyamala	0.0000	0.0000	0.0000	0.0000	0.0000	0.0000	0.0000	0.0000	0.0000
R56	Mzimuni	0.0008	0.0008	0.0008	0.0000	0.0025	0.0000	0.0025	0.0000	0.0050
R56	Ndugumbi	0.0000	0.0000	0.0000	0.0000	0.0000	0.0000	0.0000	0.0000	0.0000
**Municipality: Temeke**										
R12	Azimio	0.1510	0.1403	0.1851	0.1200	0.1906	0.0276	0.2074	0.0204	0.2228
R12	Keko	0.0045	0.0045	0.0045	0.0000	0.0112	0.0000	0.0112	0.0000	0.0180
R12	Kurasini	0.0091	0.0072	0.0190	0.0053	0.0210	0.0046	0.0494	0.0036	0.0582
R12	Miburani	0.0131	0.0104	0.0273	0.0078	0.0298	0.0070	0.0644	0.0051	0.0874
R12	Mtoni	0.0090	0.0071	0.0187	0.0048	0.0216	0.0038	0.0531	0.0028	0.0623
R23	Azimio	0.0056	0.0045	0.0117	0.0022	0.0143	0.0020	0.0363	0.0005	0.0557
R23	Keko	0.0093	0.0074	0.0194	0.0056	0.0213	0.0051	0.0472	0.0023	0.0609
R23	Kurasini	0.0097	0.0077	0.0203	0.0057	0.0223	0.0047	0.0524	0.0030	0.0639
R23	Miburani	0.0104	0.0082	0.0216	0.0060	0.0243	0.0053	0.0558	0.0035	0.0732
R23	Mtoni	0.0064	0.0051	0.0133	0.0030	0.0155	0.0025	0.0371	0.0017	0.0495
R34	Azimio	0.0087	0.0069	0.0181	0.0049	0.0203	0.0042	0.0485	0.0015	0.0635
R34	Keko	0.0054	0.0054	0.0054	0.0023	0.0101	0.0023	0.0101	0.0008	0.0124
R34	Kurasini	0.0021	0.0017	0.0045	0.0008	0.0054	0.0006	0.0135	0.0004	0.0197
R34	Miburani	0.0008	0.0006	0.0016	0.0002	0.0024	0.0000	0.0068	0.0000	0.0107
R34	Mtoni	0.0044	0.0035	0.0092	0.0023	0.0107	0.0021	0.0251	0.0012	0.0331
R45	Azimio	0.0011	0.0011	0.0011	0.0000	0.0032	0.0000	0.0032	0.0000	0.0054
R45	Keko	0.0000	0.0000	0.0000	0.0000	0.0000	0.0000	0.0000	0.0000	0.0000
R45	Kurasini	0.0023	0.0023	0.0023	0.0000	0.0053	0.0000	0.0053	0.0000	0.0075
R45	Miburani	0.0040	0.0040	0.0040	0.0000	0.0094	0.0000	0.0094	0.0000	0.0121
R45	Mtoni	0.0058	0.0046	0.0122	0.0027	0.0144	0.0023	0.0339	0.0005	0.0429
R56	Azimio	0.0014	0.0014	0.0014	0.0000	0.0042	0.0000	0.0042	0.0000	0.0070
R56	Keko	0.0009	0.0009	0.0009	0.0000	0.0028	0.0000	0.0028	0.0000	0.0047
R56	Kurasini	0.0000	0.0000	0.0000	0.0000	0.0000	0.0000	0.0000	0.0000	0.0000
R56	Miburani	0.0026	0.0026	0.0026	0.0005	0.0052	0.0005	0.0052	0.0000	0.0062
R56	Mtoni	0.0000	0.0000	0.0000	0.0000	0.0000	0.0000	0.0000	0.0000	0.0000

Disaggregating by individual wards (Fig 4) raises an important issue: the lack of a perfect match between the time and duration of each stage/survey round, and the time when each phase of the intervention was launched. Phase 1 started in March 2006 (see Fig 2), slightly after the onset of the main rainy season, and at the end of data collection in round 3 (R3). Considering that there is a biological time lag between reducing larval survival and reducing malaria transmission from human to human, and the initial programmatic challenges of launching the intervention [28,29] (augmented by the heavy rains), we expect that incidence rates estimated for R34 (in the three wards targeted with the intervention) should be the first to potentially reflect changes due to the larval control. However, each one of these three wards (first line of three graphs in Fig 4) was interviewed at a different stage, and thus exposed to the intervention for different periods of time. Between March 2006 and the beginning of interviews there were about 180, 240, and 300 days in Buguruni, Mikocheni, and Kurasini wards, respectively. Except from Buguruni, incidence rates stabilized at lower levels after R34. One peculiarity of Buguruni is the intense migration from northern areas of the country.

Phase 2 started in May 2007, a month after interviews for R5 had started, expanding larviciding application to six additional wards (second and third lines of graphs in Fig 4). Here, between May 2007 and the beginning of the interviews there were about 30 days for Mzimuni and Keko, 60 for Miburani, and 90 for Ilala. In the case of Vingunguti and Mwananyamala, however, R5 interviews started in April 2007, before the introduction of larviciding. Thus, changes in incidence rates observed in these wards for the period R45 cannot be associated with larval control.

Phase 3 started in April 2008, a month after interviews for R6 had started, expanding larviciding application to the remaining six wards (fourth and fifth lines of graphs in Fig 4). The number of days between the onset of phase 3 and R6 interviews was about 180 for Magomeni and Kipawa, 150 for Ndugumbi and Mtoni, and 60 for Mchikichini. Azimio, however, had R6 data collection between March-May 2008, and thus data for this ward were not suitable to capture any potential contribution of the larval control intervention.

A crude analysis of the estimated incidence rates by wards is also difficult due to the large diversity in social, economic, and environmental conditions. In addition, changes in the urban landscape are dynamic, reducing or augmenting the risk of malaria transmission. As a result, wards interviewed at the same period (thus subjected to same rainfall pattern), targeted by the same intervention phase, may show distinct trends in incidence rates. For example, Ndugumbi and Mtoni were both targeted during the 3^rd^ phase of larviciding, and interviewed during stage IV of each survey round (see Fig 2), but while Ndugumbi stabilized at very low incidence rates even before larval control, Mtoni did show a steep decline in rates after the introduction of larviciding (see Fig 4). Also, Mwananyamala and Vingunguti were targeted during the 2^nd^ phase of the larval control intervention, and interviews conducted during stage I of each survey round. Estimated incidence rates for both wards were among the highest for R12, reducing significantly in R23. However, while Mwananyamala maintained low rates (even before the introduction of larviciding), Vingunguti observed an increase in incidence rates in R34 and R45. Since interviews in Stage I started before the launching of phase 2 of the intervention, any impact would only be detected in R56, and a decline was indeed observed in Vingunguti by then. Distinct patterns were also observed for Magomeni and Kipawa—both interviewed in stage V of each survey round and targeted in phase 2 of the intervention. In such a complex scenario, even the consideration of a large number of variables to define control wards (to be compared with intervention wards) is a near to impossible task. Important to note, however, are the large uncertainty intervals observed in some wards (Fig 4), mainly due to sampling variability (as detailed next).

#### Sensitivity analysis

Sensitivity analysis of the incidence rate estimates by survey round (Table 6) and by ward (Table 7) was assessed through variation intervals that reflect uncertainty from non-identifiability of the mixture model, and variation intervals that combine both non-identifiability and sampling variation. Results indicate that variation from non-identifiability of the mixture model here proposed was rather small, as we would expect considering the rigorous context-based constraint conditions that we assumed. Thus, the bulk of the uncertainty in *r*_1_ could be attributed to sampling variability. Considering the level of detail used for the estimation of incidence rates by ward (15 wards * 5 pairs of consecutive survey rounds = 75 estimates), the fact that individuals who tested positive for a malaria infection were treated with anti-malarials, and the decline in transmission observed in the UMCP (even before the larviciding intervention was launched), implied that the small counts for *n*_21_, *n*_22_, and *n*_12_ (Table 1) that were observed in some pairs of survey rounds were not unexpected.

Thus, sampling variation becomes rather important, especially for *p*_2_. The magnitude of uncertainty was smaller for the estimates by stage of the survey round since data were more aggregated (5 stages * 5 pairs of consecutive survey rounds = 25 estimates), and thus larger counts for *n*_21_, *n*_22_, and *n*_12_ (Table 2) obtained.

The largest variation intervals in estimated rates were observed in three wards in R12 that recorded particularly high counts for *n*_12_ and thus lower values for *p*_1_; Azimio had *p*_1_ = 0.368 (and thus *p*_2_ > *p*_1_ and *v*_1_ − *v*_2_ is small), Vingunguti had *p*_1_ = 0.542 (and *v*_1_ − *v*_2_ is small), and Mwananyamala had *p*_1_ = 0.603 (and *v*_1_ − *v*_2_ is small).

#### Comparison of incidence and prevalence rates

**Fig 5:**
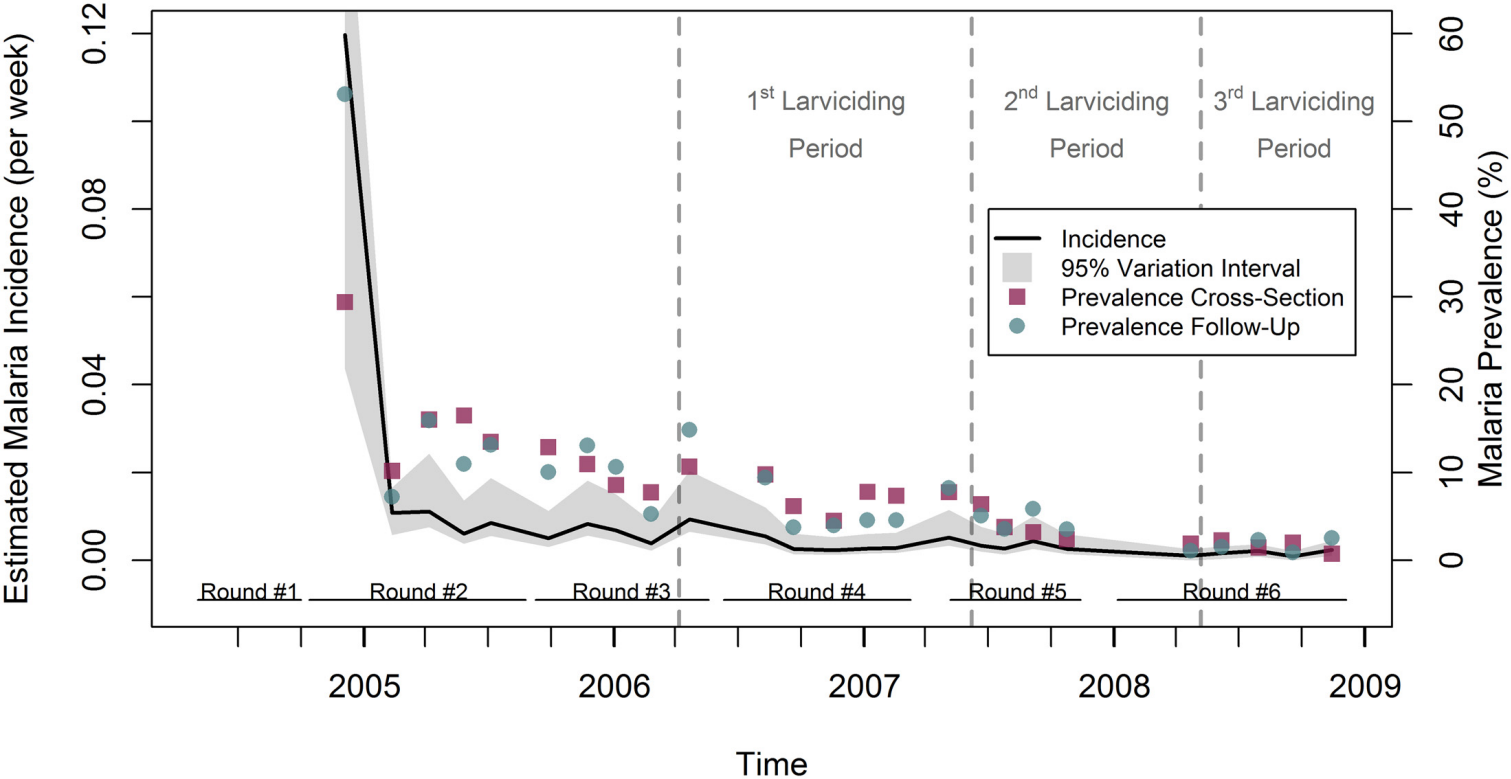
Estimated incidence rates, variability intervals, and prevalence of malaria infection by stages of the survey rounds. Variability intervals of the incidence rates consider the uncertainty due to sampling and partial identifiability of the mixture model. Prevalence rates were calculated using two distinct sets of data. First, the longitudinal data also utilized for the calculation of incidence rates (Prevalence—Follow-Up). Second, cross-sectional data collected at the same time and wards of the longitudinal data, but in different tencell units (Prevalence—Cross-Section). Each survey round of the UMCP conducted interviews in three wards at a time, or stages. Thus, each round had five stages in order to cover all 15 wards.

We compared estimated incidence rates with prevalence rates calculated directly from survey data. Since the UMCP collected both longitudinal and cross-sectional data [5], we were able to calculate two sets of prevalence rates: one utilizing the follow-up data (the same data used to calculate incidence rates), and another utilizing the cross-sectional data collected at the same time of the follow-up data, but in different ten-cell units. Fig 5 shows that, overall, prevalence rates followed the same pattern of the incidence rates, becoming much lower and more stable after phase 3 of the intervention. The same was observed when analyzing the rates disaggregated by wards (Fig 4). The two prevalence rates were similar in magnitude, with only three exceptions: Azimio, Vingunguti, and Mwananyamala, at the time when incidence rates were also extremely high.

It was reassuring that prevalence rates calculated from follow-up and cross-sectional data had similar values (with a few exceptions when the intensity of transmission was high). Despite possible intra-ward variability in malaria transmission, the ten-cell units sampled for follow-up and during cross-sectional surveys seem to be representative of the transmission pattern in the ward, which is reflected in similar prevalence rates for both data types.

#### Estimated incidence rates based on PCR adjustments

The same pattern seen for estimated incidence rates based on microscopy data was observed for PCR-adjusted incidence rates (S1 Fig). Prior to the final survey rounds (R56) of data collection, the estimated PCR-adjusted rates were slightly higher than those estimated based on microscopy data. However, as larviciding was scaled-up, estimated PCR-adjusted incidence rates near the end of the program did not differ qualitatively from what was calculated based on microscopy. Nevertheless if microscopy were to indicate an incidence rate of zero—consistent with elimination—the PCR rates being positive but small would not support such a claim.

## Discussion

The methodology we introduced for estimating incidence rates from 2-wave panel data satisfying trace $\hat{P}\left(\text{Δ}\right)<1$ utilized 2-component mixtures of continuous-time Markov chains as a class of models to represent such data. This was accompanied by an unavoidable imposition of inequality constraints to facilitate parameter identification or, in some instances, partial identification. The application of this strategy for estimation of incidence rates in an urban malaria control initiative in Dar es Salaam represented a practical instance where such tools played a decisive role in quantifying reductions in malaria acquisition rates in a low endemicity setting. With this general methodology and particular example in hand, several further issues regarding incidence rate estimation require clarification.

### a) Alternative specification of waiting time distributions

The continuous-time Markov chains all have exponentially distributed waiting time distributions in each state, which implies that their hazard rates are constant. Two-wave panel data, assumed to be generated by some 2-state continuous-time stochastic process, is not sufficiently rich to provide a basis for testing this hypothesis. However, several basic facts about malaria in diverse ecosystems make this assumption untenable. For example, in the Garki study [3], persons who survive repeated episodes of malaria in infancy and childhood have antibody titers that ensure an increasing hazard rate in the infected state—i.e. the longer an individual has detectable parasites, the more likely he/she is to clear parasites free of any intervention, and return to the uninfected state. For uninfected individuals at the end of a dry season, the hazard rate for onset of a new infection is also increasing, corresponding to the propensity for rain and, thereby, standing water. One of many possible parameterizations of these qualitative ideas is given by the waiting time distributions *F*^(*i*)^(*t*), *t* > 0, where *i* = 1,2 designate the states uninfected (*i* = 1) and infected (*i* = 2), and having probability density functions

$$f_{i}\left(t\right)=\frac{\beta_{i}^{\alpha_{i}}t^{\alpha_{i}-1}e^{-\beta_{i}t}}{\Gamma \left(\alpha_{i}\right)},\;\alpha_{i},\;\beta_{i},\;t>0\text{ for }i=1,2$$ (7)

and hazard rates $h_{i}\left(t\right)=\frac{f_{i}\left(t\right)}{1-F^{\left(i\right)}\left(t\right)},\;i=1,2.$ Here *h*_*i*_(*t*) is: increasing if *α*_*i*_ > 1, constant if *α*_*i*_ = 1, and decreasing if *α*_*i*_ < 1.

The family of Gamma densities eq (7) are the basis for obtaining an expression for *P*(Δ) within the class of semi-Markov models by solving the backward integral equation system [30].

$$p_{ij}\left(0,t\right)=\delta_{ij}\left[1-F_{i}\left(t\right)\right]+\sum_{k=1}^{r}\int_{0}^{t}f_{i}\left(s\right)m_{ik}p_{kj}\left(0,t-s\right)ds$$ (8)

where *δ*_*ij*_ = 1if *i* = *j*, *δ*_*ij*_ = 0 if *i* ≠ *j*, and 1 ≤ *i*, *j* ≤ *r* with *M* = ‖*m*_*ik*_‖ an r x r stochastic matrix having *m*_*ii*_ = 0 for 1 ≤ *i*, *j* ≤ *r*.

Specification eq (8) holds for general r-state process and waiting time densities *f*_*i*_(*t*), 1 ≤ *i* ≤ *r*. For our purposes, *r* = 2, *m*_12_ = *m*_21_ = 1, and we focus on the 2-parameter family eq (7). Here we set *t* = Δ and identify *p*_*ij*_(0,Δ) with the (*i*,*j*) entry in *P*(Δ).

The equation system eq (8) is amenable to the following interpretation: (i) when *i* ≠ *j*, *p*_*ij*_(0,*t*) consists of the sum of products of three factors: the probability of a first departure from state *i* at time *s*, the probability of a transition from state *i* to state *k* at that instant, and the probability of transferring to state *j* by some combination of moves in the interval *t*−*s* The summation is over all intermediate states *k* and all time divisions *s* in (0, *t*); (ii) when *i* = *j*, in addition to the preceding term, there is the probability of not transferring out of state *i* during (0, *t*). This is given by the first term.

With empirical data, solving the system of equations ${\hat{p}}_{i,j}\left(\text{Δ}\right)=p_{ij}\left(0,\text{Δ}\right),\;i=1,2$ for parameter values as in the Gamma specification above, requires a priori context-dependent constraints on the parameters—to secure identification or partial identification—and numerical inversion calculations in eq (8). Going back to the empirical analyses in the Garki baseline surveys [3], the disconcerting issue that now arises is that the entire set of 2-wave panel data sets shown in Singer & Cohen [7], with incidence and recovery rates computed within the class of continuous time Markov chain models, could just as well have been used to estimate incidence and recovery rates within the class of 2-state semi-Markov models with Gamma distributed waiting times. The same can, of course, be said for the rates computed in the prior section for Dar es Salaam now using, in some of the trace *P* < 1 cases, 2-component mixtures of semi-Markov models with analogous inequality constraints facilitating identification or partial identification of parameters.

### b) In low-endemicity settings, do we need to take formal account of the possibility of many (i.e., 3 or more) unobserved transitions?

If we use a crude incidence rate given by $r_{1}{}^{\left(crude\right)}=\frac{{\hat{p}}_{12}\left(\text{Δ}\right)}{\text{Δ}}$, this essentially assumes that there is at most one unobserved transition in the inter-survey interval, Δ. For the Garki surveys where Δ = 10 weeks, we find, not surprisingly, that *r*_1_^(*crude*)^<*r*_1_^(*Markov*)^ in the baseline surveys. In the lower endemicity setting of Dar es Salaam, the same inequality holds when trace $\hat{P}>1$, but now Δ ≈ 40 weeks. If Δ had been approximately 10 weeks for Dar es Salaam, we would anticipate very little difference between *r*_1_^(*crude*)^ and *r*_1_^(*Markov*)^.

We also find that *r*_1_^(*crude*)^ < *r*_1_^(*Mixture*)^ when trace $\hat{P}<1$ in Dar es Salaam, but this is decidedly influenced by the long inter-survey intervals. As two examples, consider the ward Buguruni, for survey intervals R23 (with Δ = 42.2 weeks) and R56 (with Δ = 49.2 weeks). For R23, *r*_1_^(*crude*)^ = 0.0031 and *r*_1_^(*Mixture*)^ = 0.0081. For R56, *r*_1_^(*crude*)^ = 0.0007 and *r*_1_^(*Mixture*)^ = 0.0039. Thus, during the last survey interval, R56, when the larvicide intervention was operating effectively, we still have *r*_1_^(*crude*)^ and *r*_1_^(*Mixture*)^ differing by a factor of 5.6. Taking formal account of the possibility of multiple unobserved transitions clearly makes a difference, and is preferable to the crude incidence rate, generally.

### c) Comparison of incidence rates and incidence defined as *Inc* = [Number of positive species-specific clinical cases observed during the duration of a survey]/[(Number of people observed over the survey duration) x (duration of survey)] [2]

For the 2-wave panel data in Dar es Salaam, we have:

$$Inc=\frac{n_{22}+n_{21}+n_{12}}{N*\text{Δ}}$$ (9)

where *N* = *n*_11_+*n*_12_+*n*_21_+*n*_22_. Using eq (9) we compare *Inc* with *r*_1_^(*Mixture*)^ for two wards, Buguruni and Kurasini, early in the larviciding program, R23, and at the end of it, R56 (Table 8).

**Table 8:** Comparison of incidence (*Inc*) and incidence rates for Buguruni and Kurasini wards.

Survey Interval	Ward	*Inc*	*r*_1_^(*Mixture*)^
R23	Buguruni	0.0068	0.0081
	Kurasini	0.0095	0.0100
R56	Buguruni	0.0034	0.0039
	Kurasini	0.00032	0

The general lesson here is that *Inc* < *r*_1_^(*Mixture*)^ except for survey rounds where there are almost no infected cases. Indeed, for Kurasini at R56 we have a raw table of counts given by $\left(\begin{array}{cc} n_{11} & n_{12}\\ n_{21} & n_{22} \end{array}\right)=\left(\begin{array}{cc} 63 & 0\\ 1 & 0 \end{array}\right)$. Here there is no apparent transmission between survey rounds 5 and 6. The infected individual at survey is, in accordance with the study protocol, treated with anti-malarial drugs. The general inequality *Inc* < *r*_1_^(*Mixture*)^ is basically a consequence of the fact that unobserved transitions are not taken into account in *Inc*.

In moderate to high transmission areas, we anticipate that *Inc* will be substantially downward biased as a result of lack of formal consideration of unobserved transitions.

### d) Estimating incidence from single and multiple prevalence surveys

Different methods have been proposed to generate incidence (as defined in this paper, or the rate of occurrence of an event) from prevalence data [31–34]. As more applications of the methodology here introduced are made, analysis that would produce both incidence and prevalence rates could provide a unique opportunity to evaluate the best approach to obtain incidence rate estimates from prevalence rates—currently, it is unclear what is the best strategy. Such an exercise could lead to clear recommendations that would have both a wide applicability in malaria endemic countries and a crucial importance for National Malaria Control Programs (e.g., planning and evaluation of the cost-effectiveness of interventions).

In conclusion, this paper introduced new methodology for estimating incidence rates from 2-wave panel data with interval censoring, which is applicable in the many cases where the extant Markovian models are inapplicable. The methodology is suitable to settings with any malaria transmission level, given the availability of longitudinal data. In addition, we present a strategy for quantifying the uncertainty in estimation of incidence rates. It is hereby distributed with a well-documented programming code that allows the use of the method in R software.

## Supporting Information

S1 Code R-code to estimate malaria incidence rates from interval-censored longitudinal data, and to calculate variability intervals for the estimated rates. This file contains a commented version of the code written in R. The.R file is also available for download (S2 Code).
(DOCX)

S2 Code R-code to estimate malaria incidence rates from interval-censored longitudinal data, and to calculate variability intervals for the estimated rates. This file contains the.R file (which can be directly read and used in R).
(R) Click here for additional data file.

S1 Fig Estimated incidence rates and PCR-adjusted incidence rates, and partial identifiability intervals by wards and consecutive pairs of survey rounds. Each column of graphs shows wards from one municipality: the first column has wards from Kinondoni, the second column those from Temeke, and the third column shows wards from Ilala. Wards in the first line of the Figure were included in the first phase of the intervention; second and third line of graphs include wards targeted during the second phase of the intervention; and the last two lines show the wards included in the third and last phase of the intervention. The line indicating the onset of the larval control was placed on the pair of survey round at which any impact of each phase of the larval control could be observed. The Roman number after the name of the ward indicates the stage when interviews were conducted in each survey round. Assumptions for the calculation of PCR-based rates were extracted from Okell et al. [36].
(TIFF)
