# Supplementary material for: Malaria Incidence Rates from Time Series of 2-Wave Panel Surveys
Source: PLoS Comput Biol. 2016 Aug 10;12(8):e1005065. doi: 10.1371/journal.pcbi.1005065 (PMC4980052; doi:10.1371/journal.pcbi.1005065)
Supplement: S1 Code — This file contains a commented version of the code written in R. The.R file is also available for download (S2 Code). (DOCX) [file pcbi.1005065.s001.docx]

**R-code to estimate malaria incidence rates from interval-censored longitudinal data**

Here we present a commented version of the code written in R. The .R file is also available for download (S2Code).

Incidence <- function(Data, TimeInt, Eps=0.008, Limit_u1=0.2, Value_u2=1, Limit_s1=0.5, Grid=NULL, N=100, ReturnAll=FALSE){

# Function **Incidence()** estimates incidence rates using continuous-time mixture Markov chains, as described in Castro, Maheu-Giroux, Chiyaka, and Singer (2016). It requires the following inputs:

# **Data**: Vector of the form c(n11, n12, n21, n22) that contains the raw count of elements of a 2x2 matrix, such as: n11 is the number of non-infected individuals in the 1st survey round who remained not infected in the 2nd round; n12 is the number of non-infected individuals in the 1st survey round who were infected in the 2nd round; n21 is the number of infected individuals in the 1st survey round who were not infected in the 2nd round; and n22 is the number of infected individuals in the 1st survey round who remained infected in the 2nd round. From these counts, p1=n11/(n11+n12) and p2=n22/(n21+n22). This vector should be created from the longitudinal data.

# **TimeInt**: Time interval between two consecutive survey rounds. This is calculated considering the date of interview of each survey round (ideally day, month and year).

# **Eps**: The espilon value used to maximize (v1-v2), and it is required to be small.

# **Limit_u1:** Upper bound for the setting-specific constraint on u1. Set to 0.2 by default.

# **Value_u2**: Value for u2. Default is set to 1.

# **Limit_s1:** Upper bound (excluding value) for the setting-specific constraint on s1. Set to s1<0.5 by default.

# **Grid**: Optional argument supplying the grid of point covering the parameter space for (u1, s1). This argument is used mostly internally to speed up computation when performing the probabilistic sensitivity analysis.

# **N**: Number of equally-spaced values of u1 to sample on the curve of partial identifiability.

# **UncertaintyInterval**: Internal argument used to indicate that the output of this function should be all sampled values of incidence (r1). To be used with the **Sensitivity()** function.

# **Incidence()** returns a vector with the median (Median), 2.5th percentile (LCI), 97.5th percentile (UCI), minimum (Min) and maximum (Max) of the incidence rates estimated.

# Pre-processing step of the vector to obtain the 2x2 matrix P.

TwoByTwo <- t(matrix(as.numeric(Data), ncol=2, nrow=2))

Totals <- rowSums(TwoByTwo)

TwoByTwo_p <- TwoByTwo/Totals

TwoByTwo_p <- ifelse(is.na(TwoByTwo_p), 0, TwoByTwo_p)

# When dividing 0/n22, NA are produced. The next command line replaces NA with 0.

P1 <- TwoByTwo_p[1,1]

P2 <- TwoByTwo_p[2,2]

# If (p1, p2) is in the interior of the unit square.

if(P1>0 & P1<1 & P2>0 & P2 <1){

# Create a grid of parameter sets for (u1, s1) that covers the whole parameter space.

if(is.null(Grid)){

# The objective is to set good coverage of points in the u1 x s1 plane.

# Since u1=0 implies that the incidence rate, r1=[infinity], the lower bound is set to 0.00005.

grid <- NULL

grid$u1 <- seq(from=0.00005, to=Limit_u2, by=0.001)

grid$s1 <- seq(from=0, to=(Limit_s1 – 1e-10), by=0.001)

# After selecting points at regular intervals along u1 and s1, the grid of points is expanded to cover the whole parameter space.

Grid <- expand.grid(grid$u1, grid$s1)

colnames(Grid) <- c('u1','s1') }

v1 <- (P1 - Grid$s1*Grid$u1)/(1 - Grid$s1)

# Only consider the values of v1 that lie in (0, 1).

v1 <- na.omit(ifelse(v1>=0 & v1<=1, v1, NA))

# Take the largest value of v1, call it v1x, and use it as a fixed parameter in the mixture representation.

# Once v1x has been set, there are two possibilities:

# 1) There is a unique pair (u1, s1) that correspondents to v1x, in which case the model is exactly identified.

# 2) (u1, s1) that correspond to the fixed parameter v1x lies on the curve of (u1, s1).

v1x <- max(v1)

# Next, calculate v2 and s2.

v2x <- 1 + Eps - v1x

s2 <- (P2 - v2x)/(Value_u2-v2x)

# Check that s2 lies in the interval [0, 1).

# If it is not the case, increase it using the **Eps** parameter, until s2 lies in that interval.

if (s2<0) {

Small <- seq(from=0.001, to=1, by=0.001)

s2 <- (P2 - v2x+Small)/(Value_u2-v2x+Small)

v2p <- v2x + Small[min(which(s2>0))]

Epsx <- Eps + (v2p - v2x)

v2x <- v2p

Eps <- Epsx }

# The extent of non-identifiability of the mixture model can now be represented as the set of points (u1, s1) on the curve defined by (u1*s1 - s1*v1x)=(p1 - v1x).

# First determine the range of plausible values given v1x and the other constraints.

Values <- NULL

Values$u1 <- seq(from=0.00005, to=Limit_u1, by=0.0005)

Values$s1 <- (P1 - v1x) / (Values$u1 - v1x)

# Verify that s1 is in the (0, 0.5) interval and, if that is not the case, reject these samples.

Values$s1 <- ifelse(Values$s1>=0 & Values$s1<Limit_s1, Values$s1, NA)

# The vector named **Values** below has the range of plausible u1 values to be sampled from.

Values <- na.omit(as.data.frame(Values))

# Generate N equally spaced samples of u1 for the range of plausible values of u1, conditional on v1x.

NI <- NULL

BY <- (max(Values$u1) - min(Values$u1))/N

NI$u1 <- seq(from=min(Values$u1), to=max(Values$u1), by=BY)

NI$s1 <- (P1 - v1x) / (NI$u1 - v1x)

# Verify that s1 is in the [0, 0.5) interval and, if that is not the case, reject these samples.

NI$s1 <- ifelse(NI$s1>=0 & NI$s1<Limit_s1, NI$s1, NA)

NI <- na.omit(as.data.frame(NI))

# Estimate the incidence rate.

r1 <- NI$s1*(log(NI$u1 + Value_u2 - 1)/(NI$u1 + Value_u2 -2))*((1 - NI$u1)/TimeInt) + (1 - NI$s1)*(log(Eps)/(Eps - 1))*((1 - v1x)/TimeInt)

# The reported estimates correspond to the median, 2.5^th^ percentile, 97.5^th^ percentile, minimum, and maximum values of estimated incidence rates.

CI <- quantile(r1, probs=c(0.5, 0.025, 0.975))

Inc <- data.frame(Median=CI[1], LCI=CI[2], UCI=CI[3], Min=min(r1), Max=max(r1)) }

# If (p1, p2) is on the boundary of the unit square, special consideration must be put forth.

if(P1>0 & P1<1 & P2==0){

# When p=(p1, 0), p1 and the constraints pertaining to (v1, v2) uniquely determine r1.

r1 <- log(Eps)*(1 - P1)/((Eps - 1)*TimeInt)

Inc <- data.frame(Median=r1, LCI=r1, UCI=r1, Min=r1, Max=r1)}

# If P1=0, we can’t estimate incidence

if(P1==0){

r1 <- NA

Inc <- data.frame(Median=r1, LCI=r1, UCI=r1, Min=r1, Max=r1)}

# If P1=1, incidence is zero.

if(P1==1){

r1 <- 0

Inc <- data.frame(Median=r1, LCI=r1, UCI=r1, Min=r1, Max=r1)}

#When P2=1, we do:

if(P1>0 & P1<1 & P2==1){

r1 <- -log(P1)/TimeInt

Inc <- data.frame(Median=r1, LCI=r1, UCI=r1, Min=r1, Max=r1)}

if(ReturnAll==TRUE){ Inc <- r1 }

return(Inc)}

# --- End of function ---

# Examples:

# Magomeni ward, survey rounds 1 and 2 (R12).

MAG <- c(78, 7, 50, 8)

Incidence(MAG, TimeInt=42.11)

# Vingunguti ward, R12.

VIN <- c(32, 27, 2, 2)

Incidence(VIN, TimeInt=29.11)

**The function below calculates malaria incidence rates adjusting for sub-microscopic infections not detected by microscopy**

Incidence_PCR <- function(Data, TimeInt, Eps=0.008, Limit_u1=0.2, Value_u2=1, Limit_s1=0.5, Grid=NULL, N=100){

# Function **Incidence_PCR()** estimates incidence rates, while adjusting for the submicroscopic infections not detected by microscopy, using continuous-time mixture Markov chains, as described in Castro, Maheu-Giroux, Chiyaka, and Singer (2016). It requires the following inputs:

# **Data**: Vector of the form c(n11, n12, n21, n22) that contains the raw count of elements of a 2x2 matrix, such as: n11 is the number of non-infected individuals in the 1st survey round who remained not infected in the 2nd round; n12 is the number of non-infected individuals in the 1st survey round who were infected in the 2nd round; n21 is the number of infected individuals in the 1st survey round who were not infected in the 2nd round; and n22 is the number of infected individuals in the 1st survey round who remained infected in the 2nd round. From these counts, p1=n11/(n11+n12) and p2=n22/(n21+n22). This vector should be created from the longitudinal data.

# **TimeInt**: Time interval between two consecutive survey rounds. This is calculated considering the date of interview of each survey round (ideally day, month and year).

# **Eps**: The epsilon value used to maximize (v1-v2), and it is required to be small.

# **Limit_u1:** Upper bound for the setting-specific constraint on u1. Set to 0.2 by default.

# **Value_u2**: Value for u2. Default is set to 1.

# **Limit_s1:** Upper bound (excluding value) for the setting-specific constraint on s1. Set to s1<0.5 by default.# **Grid**: Optional argument supplying the grid of point covering the parameter space for (u1, s1). This argument is used mostly internally to speed up computation when performing the probabilistic sensitivity analysis.

# **N**: Number of equally-spaced values of u1 to sample on the curve of partial identifiability, default set to 100.

# **Incidence_PCR()** returns a vector with the median (Median), 2.5th percentile (LCI), 97.5th percentile (UCI), minimum (Min) and maximum (Max) of the incidence rates estimated, adjusting for the missed sub-microscopic infections. (These intervals do not take into account sampling variability).

# Internal functions (logit and inverse logit)

logit <- function(x) {log(x/(1-x))}

invlogit <- function(x) {exp(x)/(1+exp(x))}

# Pre-processing step of the vector to obtain the 2x2 matrix P.

TwoByTwo <- t(matrix(as.numeric(Data), ncol=2, nrow=2))

Totals <- rowSums(TwoByTwo)

TwoByTwo_p <- TwoByTwo/Totals

TwoByTwo_p <- ifelse(is.na(TwoByTwo_p), 0, TwoByTwo_p)

# When dividing 0/n22, NA are produced. The next command line replaces NA with 0.

p1 <- TwoByTwo_p[1,1]

p2 <- TwoByTwo_p[2,2]

# The coefficients from regression to predict the prevalence ratio (Okell et al. 2012. Nature Communications). (Note that we did not take into account the uncertainty in the estimation of the regression coefficient.)

int <- 0.95402 # Intercept

b1 <- -0.1316 # Coefficient

# Observed prevalence (microscopy) at first wave

P_t0 <- Totals[2]/sum(Totals)

# If there are no observed events, we replace the observed prevalence by the upper bound of the 25% confidence interval.

if (Totals[2]==0) { P_t0 <- binom.test(0, sum(Totals), conf.level=0.25)$conf.int[2] }

# Observed prevalence (microscopy) at second wave

P_t1 <- colSums(TwoByTwo)[2]/sum(Totals)

# If there are no observed events, we replace the observed prevalence by the uper bound of the 25% confidence interval.

if (colSums(TwoByTwo)[2]==0) { P_t1 <- binom.test(0, sum(Totals), conf.level=0.25)$conf.int[2] }

# Unobserved PCR prevalence at first wave

Pt0 <- invlogit(int + logit(P_t0)*(1+b1))

# Unobserved PCR prevalence at second wave

Pt1 <- invlogit(int + logit(P_t1)*(1+b1))

# Because we are losing the longitudinal component of the data by adjusting for sub-microscopic infections, we need to recreate the potential 2*2 tables.

n11_n22 <- sum(Totals) - sum(Totals)*Pt0 - sum(Totals)*Pt1

TbT <- data.frame(N11=0:sum(Totals), N22=NA)

TbT$N22 <- TbT$N11 - n11_n22

TbT$n11 <- TbT$N11

TbT$n12 <- sum(Totals)*(1-Pt0) - TbT$N11

TbT$n22 <- TbT$N2

TbT$n21 <- sum(Totals) - TbT$n11 - TbT$n12 - TbT$N22

TbT$Total <- TbT$n11 + TbT$n12 + TbT$n22 + TbT$n21

TbT$P1 <- TbT$n11/(TbT$n11+ TbT$n12)

TbT$P2 <- TbT$n22/(TbT$n21+ TbT$n22)

# We remove all potential 2*2 tables that are either implausible or that do not meet the following condition:

Condition <- ifelse(p1>p2, 1, 0)

if (Condition==1){

TbT$ToRemove <- ifelse(TbT$P1>TbT$P2 & TbT$P1<=1 & TbT$P2<=1 & TbT$n22>=0 & TbT$n12>=0 & TbT$n21>=0, 1, NA)}

if (Condition==0){

TbT$ToRemove <- ifelse(TbT$P1<=1 & TbT$P2<=1 & TbT$n22>=0 & TbT$n12>=0 & TbT$n21>=0, 1, NA)}

TbT <- na.omit(TbT)

New2by2 <- data.frame(TbT$n11, TbT$n12, TbT$n21, TbT$n22)

# We calculate the incidence for these adjusted 2*2 tables using the Incidence() function.

r1 <- NULL

for (i in 1:dim(New2by2)[1]){

r1.i <- Incidence(Data=New2by2[i,], TimeInt=TimeInt, Eps=Eps, Limit_u1=Limit_u1, Value_u2=Value_u2, Limit_s1=Limit_s1, Grid=Grid, N=N, ReturnAll=TRUE); r1.i

r1 <- c(r1, r1.i) }

CI <- quantile(r1, probs=c(0.5, 0.025, 0.975), na.rm=TRUE)

Inc <- data.frame(Median=CI[1], LCI=CI[2], UCI=CI[3], Min=min(r1, na.rm=TRUE), Max=max(r1, na.rm=TRUE)) }

# --- End of function ---

# Example for Magomeni ward, survey rounds 1 and 2 (R12).

MAG <- c(78, 7, 50, 8)

Incidence(MAG, TimeInt=42.11) # Without adjustment

Incidence_PCR(MAG, TimeInt=42.11) # With adjustment

# Example for Vingunguti ward, R12.

VIN <- c(32, 27, 2, 2)

Incidence(VIN, TimeInt=29.11) # Without adjustment

Incidence_PCR(VIN, TimeInt=29.11) # With adjustment

**The next function calculates variability intervals for the estimated malaria incidence rates, taking into account sampling variability (this function does not make adjustments for sub-microscopic infections).**

Sensitivity <- function(Data, TimeInt, R=1000, Eps=0.008, Limit_u1=0.2, Value_u2=1, Limit_s1=0.5, N=100, digits=6){

# Function **Sensitivity()** requires the following inputs:

# **Data**: Matrix (or vector) where each row has the raw count of the 2x2 tables in the following order: n11, n12, n21, and n22.

# **TimeInt**: Time interval between two consecutive survey rounds. This is calculated considering the date of interview of each survey round (ideally day, month and year).

# **R**: Number of draws for the sensitivity analysis due to sampling variability. Default is set to 1000.

# **N**: Number of draws of incidence on the curve of partial identifiability. Default set to 100.

# **Eps**: The epsilon value used to maximize (v1-v2), and it is required to be small.

# **Limit_u1:** Upper bound for the setting-specific constraint on u1. Set to 0.2 by default.

# **Value_u2**: Value for u2. Default is set to 1.

# **Limit_s1:** Upper bound (excluding value) for the setting-specific constraint on s1. Set to s1<0.5 by default. # **digits**: Number of decimal places for the results.

# The function **Sensitivity()** returns a vector or matrix with 10 columns:

# 'MedianPI': the median incidence computed with the function **Incidence()** using the original data matrix (only takes into account partial identifiability (PI)).

# 'LCI_PI': Lower bound of the 95% variability interval, only taking into account partial identifiability (PI).

# 'UCI_PI': Upper bound of the 95% variability interval, only taking into account partial identifiability (PI).

# 'MinPI': Minimum incidence rate consistent with partial identifiability assumptions (does not take into account sampling variability).

# 'MaxPI': Maximum incidence rate consistent with partial identifiability assumptions (does not take into account sampling variability).

# 'Median': Median incidence rates, taking into account partial identifiability and sampling variability.

# 'LCI': Lower bound of the 95% variability interval that takes into account partial identifiability and sampling variability.

# 'UCI': Upper bound of the 95% variability interval that takes into account partial identifiability and sampling variability.

# 'Min2.5': 2.5th percentile of the minimum incidence rates recorded for each of the R simulated tables (takes into account partial identifiability and sampling variability).

# 'Max97.5': 97.5th percentile of the maximum incidence rates recorded for each of the R simulated tables (takes into account partial identifiability and sampling variability).

# The syntax of the function **Sensitivity()** is detailed below:

NbTables <- dim(Data)[1]

NbTables <- ifelse(is.null(NbTables), 1, NbTables)

Results <- NULL

PB <- txtProgressBar(1, NbTables*R, style=3)

# The following loop performs the sensitivity analysis for each row of the **Data** matrix.

for (i in 1:NbTables){

if(NbTables==1) { Table.i <- t(matrix(as.numeric(Data), ncol=2, nrow=2)) }

if(NbTables>1) { Table.i <- t(matrix(as.numeric(Data[i,]), ncol=2, nrow=2))}

N1 <- rowSums(Table.i)[1]

N2 <- rowSums(Table.i)[2]

Prob1 <- ifelse(is.na(Table.i[1,1]/N1), 0, Table.i[1,1]/N1)

Prob2 <- ifelse(is.na(Table.i[2,2]/N2), 0, Table.i[2,2]/N2)

# Create a grid of points for the parameter space (u1, s1).

# By computing the grid only once for each matrix (using the argument Grid=), the computations are faster.

grid <- NULL

grid$u1 <- seq(from=0.00005, to=Limit_u1, by=0.001)

grid$s1 <- seq(from=0, to=(Limit_s1 – 1e-10), by=0.001)

Grid <- expand.grid(grid$u1, grid$s1)

colnames(Grid) <- c('u1','s1')

# Sample p1 and p2 R times from independent binomial distribution.

n11 <- rbinom(n=R, size=N1, prob=Prob1)

n22 <- rbinom(n=R, size=N2, prob=Prob2)

# Note that if Prob2 is equal to 0, all resampled values of n22 will be equal to zero.

# The following loop calculates the incidence **R** times based on the samples values of p1 and p2.

r1 <- NULL

Min <- NULL

Max <- NULL

for (j in 1:R){

TbT.j <- c(n11[j],(N1-n11[j]), N2-n22[j], n22[j])

# Using the ReturnAll argument, the **Incidence()** function will now output 100 incidence rates, computed along the curve of partial identifiability. This will combine both uncertainty due to partial identifiability and sampling variability.

r1.j <- Incidence(Data=TbT.j, TimeInt=TimeInt[i], Eps=Eps, Limit_u1=Limit_u1, Value_u2=Value_u2, Limit_s1=Limit_s1, Grid=Grid, N=N, ReturnAll=TRUE)

r1 <- c(r1, r1.j)

Min <- c(Min, min(r1.j, na.rm=TRUE))

Max <- c(Max, max(r1.j, na.rm=TRUE))

setTxtProgressBar(PB, (i*R-(R-j))) }

# Calculate the median, and 2.5th and 97.5th percentile of the estimated incidence rates as well as the 2.5^th^ percentile of the minima and the 97.5^th^ percentile of the maxima.

CI <- quantile(r1, probs=c(0.5, 0.025, 0.975), na.rm=TRUE)

Min2.5 <- quantile(Min, probs=0.025, na.rm=TRUE)

Max97.5 <- quantile(Max, probs=0.975, na.rm=TRUE)

# Invoke again the **Incidence()** function to calculate the estimates for the incidence using the original 2x2 table and accounting for partial identifiability only.

Inc <- Incidence(Data=c(Table.i[1,1], Table.i[1,2], Table.i[2,1], Table.i[2,2]), TimeInt=TimeInt[i], Eps=Eps, Limit_u1=Limit_u1, Value_u2=Value_u2, Limit_s1=Limit_s1, Grid=Grid, N=N)

Results <- rbind(Results, data.frame(MedianPI=Inc[1], LCI_PI=Inc[2], UCI_PI=Inc[3], MinPI=Inc[4], MaxPI=Inc[5],

Median=CI[1], LCI=CI[2], UCI=CI[3], Min2.5=Min2.5, Max97.5=Max97.5, Min=min(Min), Max=max(Max)))

}

colnames(Results) <- c('MedianPI','LCI_PI','UCI_PI','MinPI','MaxPI','Median','LCI','UCI','Min2.5','Max97.5',’Min’,’Max’)

return(round(data.frame(Results, row.names=NULL), digits)) }

# --- End of function ---

# Examples:

# Magomeni ward, R12.

MAG <- c(78, 7, 50, 8)

Sensitivity(Data=MAG, TimeInt=42.11, R=100)

# Vingunguti ward, R12.

VIN <- c(32, 27, 2, 2)

Sensitivity(Data=VIN, TimeInt=29.1, R=1000)
