# Supplementary figures and images for: Malaria Incidence Rates from Time Series of 2-Wave Panel Surveys
Source: PLoS Comput Biol. 2016 Aug 10;12(8):e1005065. doi: 10.1371/journal.pcbi.1005065 (PMC4980052; doi:10.1371/journal.pcbi.1005065)

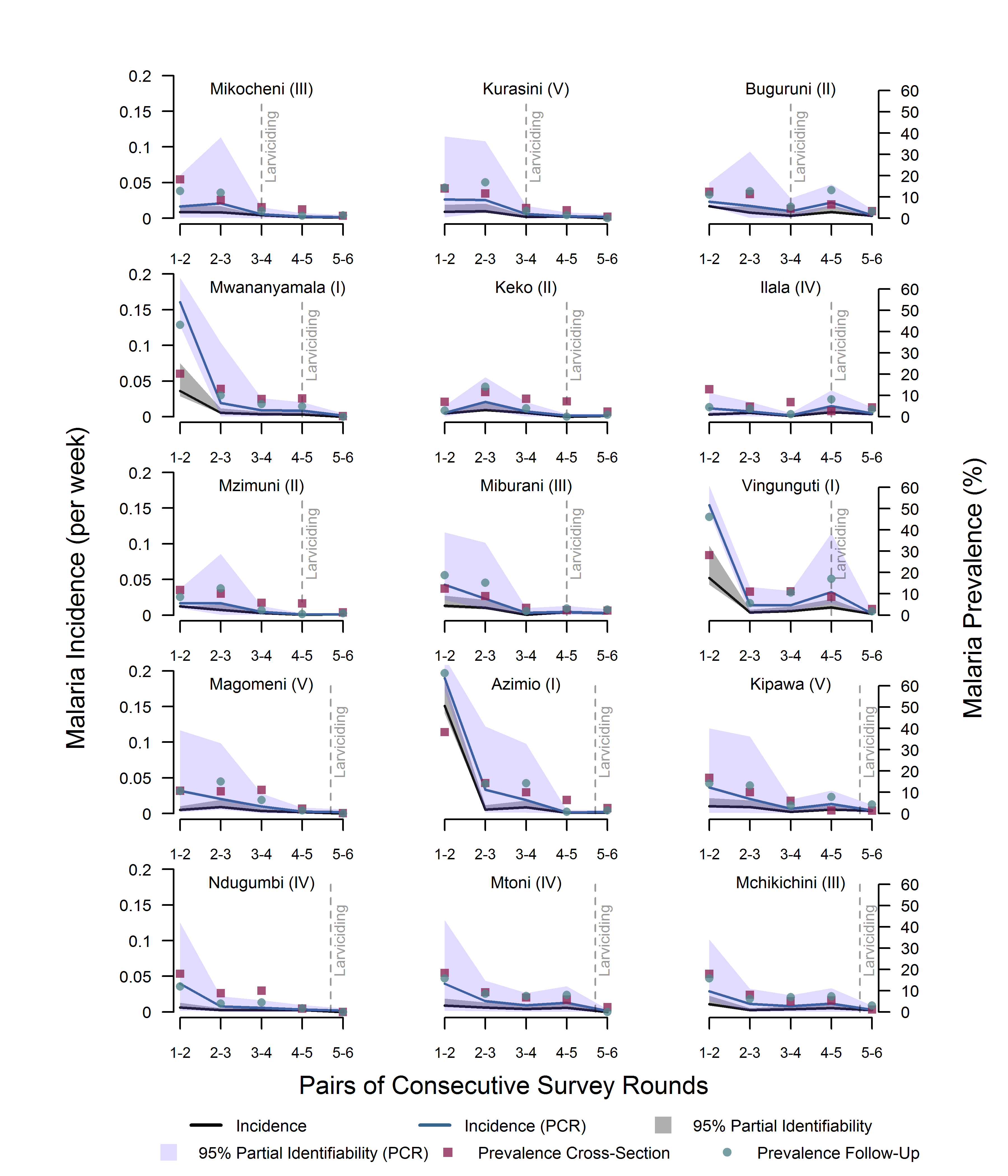

Supplement: S1 Fig — Each column of graphs shows wards from one municipality: the first column has wards from Kinondoni, the second column those from Temeke, and the third column shows wards from Ilala. Wards in the first line of the Figure were included in the first phase of the intervention; second and third line of graphs include wards targeted during the second phase of the intervention; and the last two lines show the wards included in the third and last phase of the intervention. The line indicating the onset of the larval control was placed on the pair of survey round at which any impact of each phase of the larval control could be observed. The Roman number after the name of the ward indicates the stage when interviews were conducted in each survey round. Assumptions for the calculation of PCR-based rates were extracted from Okell et al. [36]. (TIFF) [file pcbi.1005065.s003.tiff]
